# Effect of tea consumption on the development of hypertension, diabetes, and obesity: a bidirectional two-sample Mendelian randomization analysis

**DOI:** 10.3389/fnut.2024.1428445

**Published:** 2024-10-31

**Authors:** Xueying Li, Congcong Zhang, Yihui Weng, Weiming Yu, Xianlei Cai

**Affiliations:** ^1^Department of Gastroenterology, The First Affiliated Hospital, Ningbo University, Ningbo, China; ^2^Ningbo Key Laboratory of Translational Medicine Research on Gastroenterology and Hepatology, Ningbo, China; ^3^Ningbo University School of Medicine, Ningbo, China; ^4^The Metabolic Weight Loss Center, The Lihuili Affiliated Hospital, Ningbo University, Ningbo, China; ^5^Department of Gastrointestinal Surgery, The Lihuili Affiliated Hospital, Ningbo University, Ningbo, China

**Keywords:** tea consumption, hypertension, diabetes, obesity, Mendelian randomization

## Abstract

**Background:**

The effect of tea consumption on conditions such as hypertension, diabetes, and obesity has attracted significant global interest. However, the results of various studies on this topic have been mixed and somewhat contentious. Therefore, we conducted a Mendelian randomization (MR) analysis to investigate the causal relationships between tea consumption and the aforementioned health conditions.

**Methods:**

A bidirectional two-sample MR analysis was used to systematically explores the associations between tea consumption and hypertension, diabetes, and obesity. MR-Egger regression, weighted median, inverse variance weighted, and weighted mode methods were used to evaluate the potential causal associations. Leave-one-out sensitivity test was used to check the robustness of the IVW estimates.

**Results:**

MR analysis indicated that genetically predicted tea consumption is associated with a protective effect against hypertension, with an odds ratio (OR) of 0.78 and a 95% confidence interval (CI) ranging from 0.64 to 0.95. Additionally, tea consumption appeared to have a potential protective effect on type 2 diabetes and obesity related to excessive calorie intake, influenced by specific single nucleotide polymorphisms (SNPs), namely “rs57462170” and “rs17685.” No causal link was observed between the consumption of green or herbal tea and hypertension, diabetes, or obesity. However, there was a marginal negative association between type 2 diabetes and tea consumption and (OR = 0.99; 95% CI: 0.97–1.00) and a significant negative correlation between obesity due to excessive calorie intake and green tea consumption (OR = 0.35; 95% CI: 0.16–0.78).

**Conclusion:**

This study demonstrates a protective causal relationship between the consumption of tea (including black and green teas) and reduced risk of hypertension. Furthermore, our results suggest that tea intake may also have a protective effect on type 2 diabetes and obesity. The results recommend further research to verify or refine these findings.

## Introduction

Hypertension, diabetes, and obesity are widespread health concerns in contemporary society, significantly affecting individual health (1). Hypertension is identified by consistently high blood pressure, which can harm the walls of blood vessels over time and increase the burden on the heart (2), significantly heightening the risk of cardiovascular diseases like heart attacks and strokes. It can also impair kidney function, possibly leading to kidney failure (3). Diabetes, a chronic metabolic condition, is marked by persistently high blood sugar levels. This condition can damage the nervous system, leading to sensory abnormalities and pain, and affect the eyes, increasing the risk of retinopathy and blindness (4). Additionally, diabetes speeds up the process of atherosclerosis, thereby increasing the risk of cardiovascular disease and stroke (5). Obesity is a major factor contributing to hypertension and diabetes. It involves excessive fat accumulation that affects not only appearance but also places additional strain on the heart and disrupts lipid metabolism, leading to an increased risk of cardiovascular disease (6, 7). Obesity also affects insulin sensitivity, thereby increasing the risk of developing diabetes (8).

The widely accepted definition of tea includes any beverage prepared from the leaves of *Camellia sinensis*. The primary classifications of tea encompass black tea, green tea, oolong tea, and white tea. In common parlance, infusions made from flowers, herbs, or fruits are frequently called “teas,” though it is crucial to differentiate these from the traditional “tea” that originates from *Camellia sinensis* leaves. The beneficial effects of tea are conflicted and have been reported to have beneficial effects on hypertension, diabetes, and obesity (9, 10). Tea is rich in active compounds, especially polyphenols and catechins, which have antioxidant and anti-inflammatory properties that help regulate blood pressure (11). These compounds also enhance insulin sensitivity, supporting glucose metabolism and control (12, 13). Additionally, the combination of caffeine and polyphenols in tea aids in promoting fat metabolism and reducing fat accumulation (14). However, these findings are primarily based on retrospective studies, and their conclusions are still a subject of debate.

Genome-wide association studies (GWAS) use large genetic datasets from thousands of individuals to identify genetic variants associated with specific traits or diseases. This approach compares the genetic profiles of individuals with a disease to those without, pinpointing genetic variants that may increase the risk or offer protection against the disease.

Mendelian randomization (MR) is an epidemiological method recognized for its accuracy in determining causal associations between exposures and outcomes. It operates on the principle that genetic variants, serving as instrumental variables (IVs), are randomly allocated at conception, unaffected by environmental or other factors. This allows for the estimation of causal effects of exposures on outcomes, minimizing biases related to confounding or reverse causation. The combination of MR with GWAS has become increasingly popular, offering precise causal effect estimations and advancing our knowledge of complex diseases.

The current study explores the causal connections between tea consumption and the risk factors of hypertension, diabetes, and obesity through bidirectional two-sample MR analyses. The insights regarding the association of tea consumption with clinical risk factors, including hypertension, diabetes, and obesity, are expected to contribute to the development of more effective prevention and management strategies for metabolism-related diseases.

## Methods

### MR design

**Figure 1:**
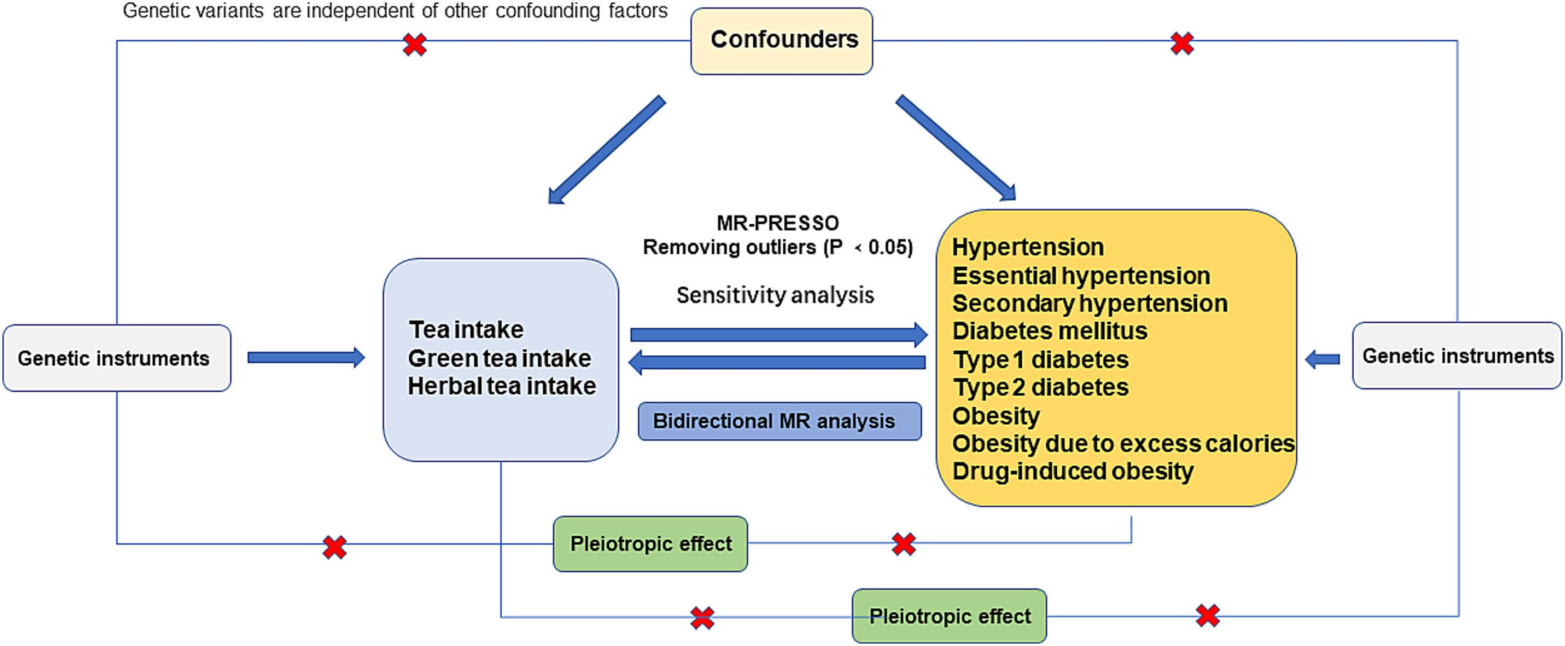
Flowchart of the data collection, processing, and analysis procedures of this study.

The STROBE-MR guidelines informed the design of this bidirectional two-sample MR study (15, 16). For MR analysis to be valid, it must satisfy three core assumptions: (1) the Relevance Assumption, which posits significant associations between genetic variants and the exposure phenotype; (2) the Independence Assumption, stipulating that the effects of genetic variants must be independent of confounding factors; and (3) the exclusion restriction assumption, asserting that the exposure of interest is the sole mediator through which genetic variants affect outcomes (17) (Figure 1). This bidirectional Mendelian randomization (MR) analysis was conducted to evaluate the impact of tea consumption quantity (including black and green teas), drinking green tea, and drinking herbal tea, both as exposures and outcomes. As an exposure, the study aimed to determine if tea consumption offers a protective effect against hypertension, diabetes, and obesity. Conversely, as an outcome, the study evaluated if individuals with hypertension, diabetes, and obesity exhibit a preference for tea consumption.

### Data sources

The datasets used in this study were publicly accessible, and all sourced publications had received ethical approval.

Tea intake–related single nucleotide polymorphisms (SNPs) were obtained from the MRC IEU OpenGWAS data infrastructure (18, 19), involving participants of European descent from the UK Biobank. The dataset for tea consumption quantity (GWAS ID “ukb-b-6066”) included 447,485 participants, measuring consumption in cups per day (mean = 3.494, standard deviation = 2.84157). Both male and female were asked: “How many cups of tea do you drink each day? (Include black and green tea).” The dataset for drinking green tea (GWAS IDs “ukb-b-4078”) consisted of 64,949 participants who were asked: “How many cups/mugs of green tea did you drink yesterday?.” The dataset for drinking herbal tea included (GWAS IDs “ukb-b-13344”) also included 64,949 participants and posed the question: “How many cups/mugs of herbal or fruit tea (infusion) did you drink yesterday?” Summary statistics for hypertension, diabetes, and obesity primarily came from the FinnGen cohort on the Integrative Epidemiology Unit (IEU) Open GWAS project (20). The FinnGen Biobank GWAS, conducted by the FinnGen team,^1^ included 55,917 patients with hypertension and 162,837 genetically matched controls. There were also 35,607 patients with diabetes mellitus and 183,185 genetically matched controls, alongside 8,908 patients with obesity and 209,827 genetically matched controls. The GWAS IDs for hypertension, essential hypertension, secondary hypertension, diabetes mellitus, type 1 diabetes, type 2 diabetes, obesity, obesity due to excess calories, and drug-induced obesity in the IEU open GWAS project were, respectively, identified as “finn-b-I9_HYPTENS,” “finn-b-I9_HYPTENSESS,” “finn-b-I9_HYPTENSEC,” “finn-b-E4_DIABETES,” “finn-b-E4_DM1_STRICT,” “finn-b-E4_DM2_STRICT,” “finn-b-E4_OBESITY,” “finn-b-E4_OBESITYCAL,” and “finn-b-E4_OBESITYDRUG” (Table 1). Additionally, the risk of type I errors in MR analysis may increase due to participant overlap. To avoid potential bias from sample overlap, the GWAS for hypertension, diabetes, and obesity based on the UK Biobank (UKB) data were excluded from this study, considering that SNPs associated with tea drinking were extracted from the UKB database.

**Table 1:** Exposures and outcomes of GWAS samples used in the study.

GWAS ID	Trait	Consortium	Sample size	Number of SNPs	Population	N of case	N of control
ukb-b-6066	Tea intake	MRC-IEU	447,485	9,851,867	European	Continuous	
ukb-b-4078	Green tea intake	MRC-IEU	64,949	9,851,867	European	Categorical	
ukb-b-13344	Herbal tea intake	MRC-IEU	64,949	9,851,867	European	Categorical	
finn-b-I9_HYPTENS	Hypertension	FinnGen biobank	218,754	16,380,466	European	55,917	162,837
finn-b-I9_HYPTENSESS	Essential hypertension	FinnGen biobank	205,694	16,380,443	European	42,857	162,837
finn-b-I9_HYPTENSEC	Secondary hypertension	FinnGen biobank	164,147	16,380,172	European	1,310	162,837
finn-b-E4_DIABETES	Diabetes mellitus	FinnGen biobank	218,792	16,380,466	European	35,607	183,185
finn-b-E4_DM1_STRICT	Type 1 diabetes	FinnGen biobank	186,323	16,380,237	European	2,649	183,674
finn-b-E4_DM2_STRICT	Type 2 diabetes	FinnGen biobank	212,351	16,380,434	European	29,166	183,185
finn-b-E4_OBESITY	Obesity	FinnGen biobank	218,735	16,380,465	European	8,908	209,827
finn-b-E4_OBESITYCAL	Obesity due to excess calories	FinnGen biobank	215,767	16,380,461	European	5,883	209,884
finn-b-E4_OBESITYDRUG	Drug-induced obesity	FinnGen biobank	209,999	16,380,447	European	115	209,884

### Selection of genetic variants

Multiple quality control measures were implemented to ensure the high quality of the instrumental SNPs. Initially, SNPs showing significant associations with exposure factors were selected based on a *p*-value criterion of less than 5 × 10^−8^. These were further refined by keeping SNPs with considerable physical distance from each other and a linkage disequilibrium threshold of r^2^ < 0.001, using data from Europeans in the 1,000 Genomes Project with a 10,000 kb window size. SNPs with a minor allele frequency (MAF) lower than 0.01 were discarded. Variability proportion was evaluated using the formula $R^{2}=2\times MAF\times \left(1-MAF\right)\times \mathrm{bet}a^{2}$ (21). The strength of SNP-outcome correlation was assessed with the F-statistic, calculated as $F=R^{2}\times \left(N-2\right)/\left(1-R^{2}\right)$ (21), with an alternative calculation method for when MAF data were unavailable: $F=\mathrm{bet}a^{2}/se^{2}$ (22). An *F*-statistic above 10 indicated a strong correlation (17).

### Statistical analysis

The stepwise methodology for MR analysis is outlined in Figure 1 through a flowchart. Initially, IVs were selected to align the GWAS data on tea consumption’s links with hypertension, diabetes, and obesity. The MR-PRESSO method was then applied to remove pleiotropic outliers before conducting the MR analysis. Subsequently, the MR-Egger regression method was used to assess horizontal pleiotropy, with a significance threshold of *p* < 0.05 indicating no evidence of horizontal pleiotropy (23). The study also explored the relationship between body mass index (BMI) and the genetic variants used as IVs, considering BMI’s significant influence on hypertension, diabetes, and obesity. The online tool PhenoScanner V2 (24) was used to check for potential confounders. Following the removal of pleiotropic SNPs, Cochran’s Q test was applied to evaluate SNP heterogeneity. The MR analysis employed various methods including weighted median, MR-Egger regression, inverse variance-weighted (IVW) model, and weighted mode (WM), with consistent results across these methods confirming the analyses’ reliability. The primary outcome was assessed using the IVW method to determine the overall causal effect of exposure on the outcome. The robustness of results was enhanced by employing three distinct analytical methods, and odds ratios (ORs) and 95% confidence intervals (CIs) were (25) calculated. A leave-one-out sensitivity analysis was also conducted to test the IVW estimates’ reliability. Moreover, GWAS data on hypertension, diabetes, and obesity were analyzed as exposures, with tea drinking as the outcome. The causal relationship was considered significant at *p* < 0.05, and the analysis was performed using the R package TwoSampleMR (v. 0.5.7).

## Results

### Baseline participant characteristics

The assessment of tea consumption was conducted across three dimensions: the total amount of tea consumed (including black and green teas), the specific intake of green tea, and the consumption of herbal tea (herbal or fruit tea). Hypertension was categorized into three groups: general hypertension, essential hypertension, and secondary hypertension. Diabetes mellitus was divided into general diabetes mellitus, type 1 diabetes, and type 2 diabetes. Similarly, obesity was classified into general obesity, obesity from excessive caloric intake, and drug-induced obesity. This detailed categorization facilitated a thorough analysis of the relationship between tea consumption and various metabolic diseases. For these 12 types of exposure, a selection of 41, 21, and 19 SNPs related to the tea consumption quantity, green tea, and herbal tea, respectively, was made. An F-statistics analysis confirmed all variances above 10, indicating a strong instrument design and affirming the study’s validity (Supplementary Table S1).

### Causal effect of tea consumption on hypertension, diabetes, and obesity

**Figure 2:**
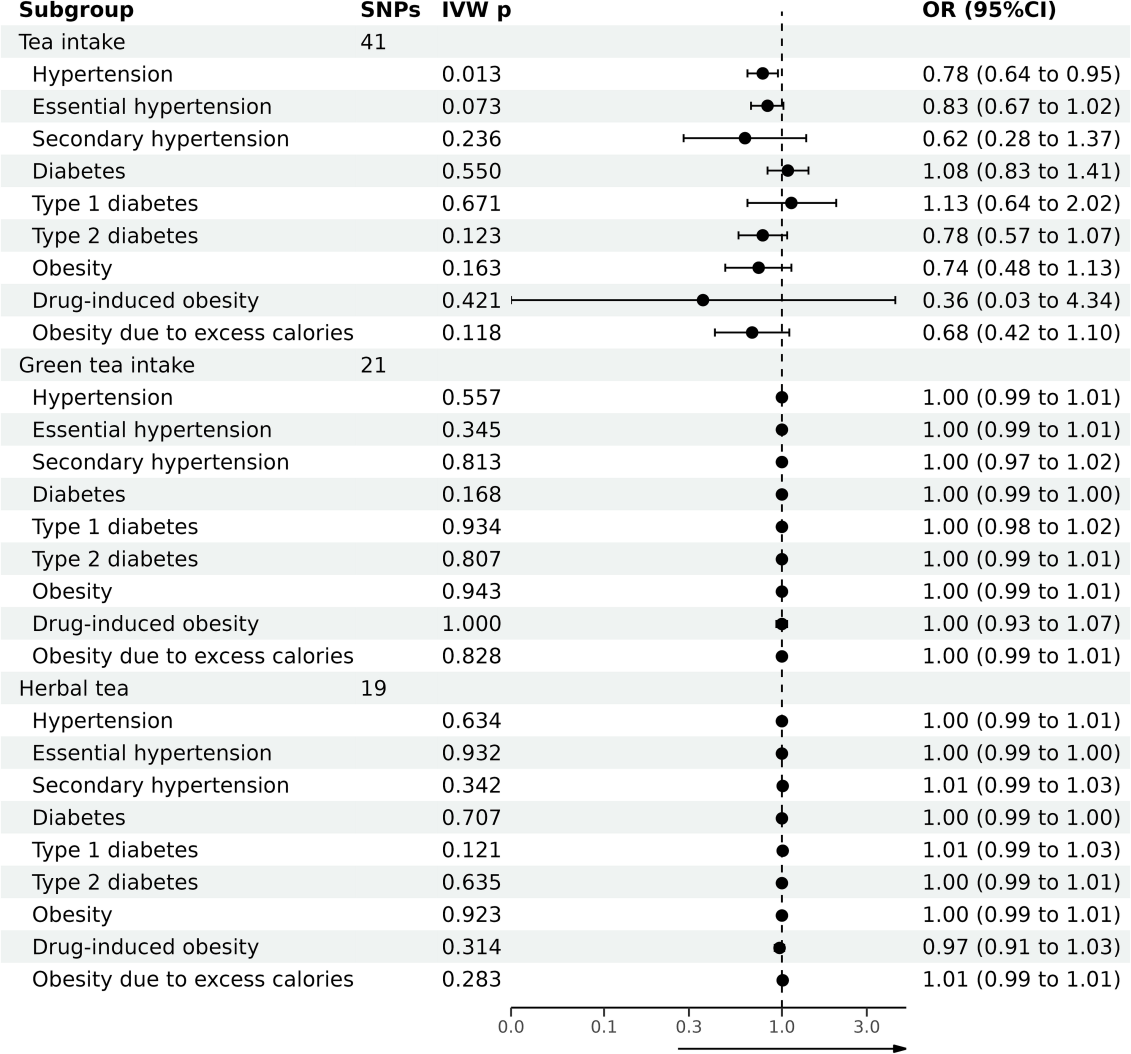
Causal effects of tea consumption on hypertension, diabetes, and obesity by MR analyses.

Table 2 summarizes the outcomes of various analytical techniques, such as MR-PRESSO, horizontal pleiotropy, and heterogeneity assessments, along with three distinct MR methods. These were used to assess the causal association of tea consumption with hypertension, diabetes, and obesity. The primary analysis was conducted using the IVW method, with the results depicted in Figure 2. Scatter plots for the MR analyses are shown in Supplementary Figure S1.

**Table 2:** MR analyses of the causal effect of tea intake on hypertension, diabetes, and obesity.

Trait	MR-Egger		WM		IVW		MR-PRESSO	Pleiotropy	Q	Heterogeneity
	OR(95%CI)	*p*	OR(95%CI)	*p*	OR(95%CI)	*p*	Global Test *P*	*P*		
Hypertension	0.85(0.55–1.32)	0.474	0.74(0.55–1.00)	0.047	0.78(0.64–0.95)	0.013	0.525	0.671	32.8	0.528
Essential hypertension	0.86(0.54–1.37)	0.530	0.69(0.50–0.95)	0.022	0.83(0.67–1.02)	0.073	0.573	0.854	31.8	0.576
Secondary hypertension	1.48(0.26–8.26)	0.661	1.01(0.33–3.10)	0.990	0.62(0.28–1.37)	0.236	0.308	0.275	42.9	0.306
Diabetes	1.66(0.79–3.52)	0.192	1.34(0.95–1.90)	0.092	1.08(0.83–1.41)	0.550	0.118	0.241	41.6	0.120
Type 1 diabetes	0.61(0.17–2.17)	0.452	0.67(0.26–1.77)	0.422	1.13(0.64–2.02)	0.671	0.456	0.291	39.0	0.472
Type 2 diabetes	0.65(0.18–2.30)	0.505	0.77(0.51–1.18)	0.233	0.78(0.57–1.07)	0.123	0.175	0.763	37.8	0.155
Obesity	0.99(0.33–2.94)	0.988	0.82(0.44–1.51)	0.518	0.74(0.48–1.13)	0.163	0.145	0.567	41.4	0.124
Drug-induced obesity	0.07(0.0003–16.6)	0.348	0.38(0.01–14.51)	0.604	0.36(0.03–4.34)	0.421	0.887	0.516	29.1	0.877
Obesity due to excess calories	1.33(0.39–4.55)	0.644	0.94(0.48–1.84)	0.867	0.68(0.42–1.10)	0.118	0.224	0.251	40.4	0.208

**Figure 3:**
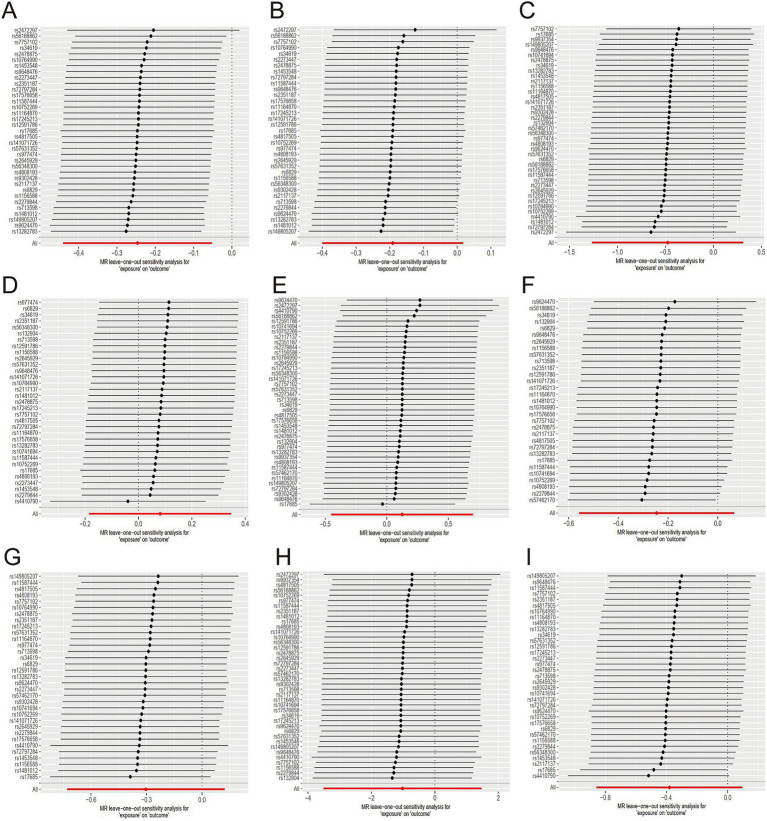
Sensitive analysis for MR analyses of the causal effect of tea intake on hypertension, diabetes, and obesity. (A) hypertension; (B) essential hypertension; (C) secondary hypertension; (D) diabetes mellitus; (E) type 1 diabetes; (F) type 2 diabetes; (G) obesity; (H) obesity due to excess calories; (I) drug-induced obesity.

The evidence indicated that genetically predicted tea consumption had a protective causal effect against hypertension, with an OR of 0.78 (95% CI: 0.64–0.95) for an increase of 2.84 cups per day. However, sensitivity analysis revealed that the SNP “rs2472297” unduly influenced the MR outcomes. Excluding this SNP eliminated the causal link between tea consumption and hypertension, as illustrated in Figure 3A. Subgroup analysis did not find a statistically significant causal relationship between tea intake and essential hypertension. However, the sensitivity analysis identified the SNPs “rs13282783,” “rs1481012,” and “rs149805207.” Removing these SNPs individually revealed a protective effect of tea on essential hypertension, shown in Figure 3B. No causal relationship was found between tea consumption and primary hypertension. The leave-one-out sensitivity test confirmed the reliability of these findings (Figure 3C).

No causal association was observed between tea consumption and diabetes (OR = 1.13; 95% CI: 0.64–2.02), including type 1 (OR = 0.78; 95% CI: 0.57–1.07) and type 2 diabetes (OR = 0.74; 95% CI: 0.48–1.13). The sensitivity test validated the consistency of the diabetes findings (Figure 3D) and type 1 diabetes (Figure 3E). Nevertheless, the analysis pinpointed the SNP “rs57462170.” Excluding this SNP revealed a protective effect of tea on type 2 diabetes, with an OR of 0.74 (95% CI: 0.55–0.99), as seen in Figure 3F.

No evidence was found to suggest that genetically predicted tea intake had a protective effect on obesity (OR = 0.74; 95% CI: 0.48–1.13), including drug-induced obesity (OR = 0.36; 95% CI: 0.03–4.34) and obesity from excess calories (OR = 0.68; 95% CI: 0.42–1.10). The sensitivity tests confirmed the robustness of the results for obesity (Figure 3G) and drug-induced obesity (Figure 3H). Interestingly, after excluding the SNP “rs17685,” a protective causal effect of tea intake on obesity due to excess calories was observed (OR = 0.62; 95% CI: 0.38–0.99; Figure 3I).

### Causal effect of green tea intake on hypertension, diabetes, and obesity

Table 3 summarizes the causal associations of green tea intake with hypertension, diabetes, and obesity. The results are graphically illustrated in Figure 2, with scatter plots for MR analyses presented in Supplementary Figure S2.

**Table 3:** MR analyses of the causal effect of green tea intake on hypertension, diabetes, and obesity.

Trait	MR-Egger		WM		IVW		MR-PRESSO	Pleiotropy	Q	Heterogeneity
	OR(95%CI)	*p*	OR(95%CI)	*p*	OR(95%CI)	*p*	Global Test *P*	*P*		
Hypertension	1.00(0.99–1.01)	0.983	1.00(0.99–1.01)	0.477	1.00(0.99–1.01)	0.557	0.835	0.718	14.4	0.811
Essential hypertension	1.00(0.99–1.01)	0.994	1.00(0.99–1.01)	0.274	1.00(0.99–1.01)	0.345	0.285	0.590	23.5	0.264
Secondary hypertension	1.01(0.96–1.06)	0.805	0.99(0.96–1.02)	0.479	1.00(0.97–1.02)	0.813	0.145	0.676	26.6	0.147
Diabetes	0.99(0.98–1.00)	0.055	1.00(0.99–1.01)	0.538	1.00(0.99–1.00)	0.168	0.183	0.143	24.4	0.141
Type 1 diabetes	1.01(0.98–1.05)	0.554	1.00(0.97–1.03)	0.954	1.00(0.98–1.02)	0.934	0.420	0.456	20.6	0.422
Type 2 diabetes	0.99(0.98–1.00)	0.184	1.00(0.99–1.01)	0.571	1.00(0.99–1.01)	0.807	0.702	0.161	11.1	0.678
Obesity	0.99(0.97–1.01)	0.305	1.01(0.99–1.02)	0.364	1.00(0.99–1.01)	0.943	0.064	0.222	30.2	0.067
Drug-induced obesity	0.95(0.83–1.10)	0.514	0.99(0.88–1.10)	0.797	1.00(0.93–1.07)	1.000	0.569	0.451	18.3	0.571
Obesity due to excess calories	0.98(0.96–1.01)	0.224	1.00(0.98–1.01)	0.715	1.00(0.99–1.01)	0.828	0.111	0.222	28.4	0.101

**Figure 4:**
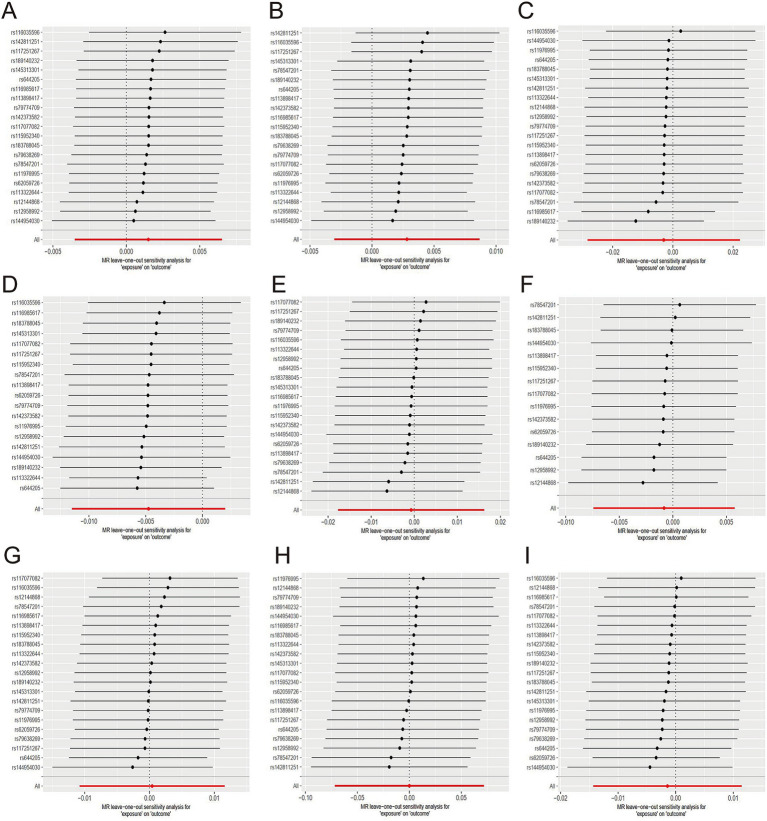
Sensitive analysis for MR analyses of the causal effect of green tea intake on hypertension, diabetes, and obesity. (A) hypertension; (B) essential hypertension; (C) secondary hypertension; (D) diabetes mellitus; (E) type 1 diabetes; (F) type 2 diabetes; (G) obesity; (H) obesity due to excess calories; (I) drug-induced obesity.

No causal relationship existed between green tea consumption and hypertension (OR = 1.00; 95% CI: 0.99–1.01), including both essential (OR = 1.00; 95% CI: 0.99–1.01) and secondary hypertension (OR = 1.00; 95% CI: 0.97–1.02). The leave-one-out sensitivity analysis confirmed the stability of these findings (Figures 4A–C).

Likewise, no causal relationship was observed between green tea consumption and diabetes (OR = 1.00; 95% CI: 0.99–1.00), encompassing type 1 diabetes (OR = 1.00; 95% CI: 0.98–1.02) and type 2 diabetes (OR = 1.00; 95% CI: 0.99–1.01). The sensitivity analysis results were consistent (Figures 4D–F).

Furthermore, no evidence indicated that genetically predicted green tea consumption causally affects obesity (OR = 1.00; 95% CI: 0.99–1.01), including drug-induced obesity (OR = 1.00; 95% CI: 0.93–1.07) and obesity due to excess calorie intake (OR = 1.00; 95% CI: 0.99–1.01). The sensitivity analysis results remained robust (Figures 4G–I).

### Causal effect of herbal tea intake on hypertension, diabetes, and obesity

Table 4 summarizes the causal association of herbal tea intake on hypertension, diabetes, and obesity. The results are graphically illustrated in Figure 2, with Scatter plots for MR analyses presented in Supplementary Figure S3.

**Table 4:** MR analyses of the causal effect of herbal tea intake on hypertension, diabetes, and obesity.

Trait	MR-Egger		WM		IVW		MR-PRESSO	Pleiotropy	Q	Heterogeneity
	OR(95%CI)	*p*	OR(95%CI)	*p*	OR(95%CI)	*p*	Global Test *P*	*P*		
Hypertension	1.00(0.98–1.02)	0.948	1.00(0.99–1.01)	0.972	1.00(0.99–1.01)	0.634	0.498	0.984	14.6	0.482
Essential hypertension	1.01(0.98–1.04)	0.494	1.00(0.99–1.01)	0.769	1.00(0.99–1.00)	0.932	0.716	0.476	10.5	0.725
Secondary hypertension	0.97(0.88–1.07)	0.609	1.01(0.99–1.03)	0.428	1.01(0.99–1.03)	0.342	0.615	0.487	16.9	0.529
Diabetes	0.99(0.96–1.01)	0.336	1.00(0.99–1.00)	0.620	1.00(0.99–1.00)	0.707	0.812	0.359	10.6	0.779
Type 1 diabetes	0.99(0.90–1.09)	0.911	1.01(0.99–1.03)	0.286	1.01(0.99–1.03)	0.121	0.076	0.693	27.0	0.079
Type 2 diabetes	0.95(0.91–1.00)	0.076	1.00(0.99–1.01)	0.754	1.00(0.99–1.01)	0.635	0.054	0.063	20.3	0.026
Obesity	1.00(0.95–1.05)	0.909	1.00(0.99–1.01)	0.982	1.00(0.99–1.01)	0.923	0.139	0.922	24.2	0.148
Drug-induced obesity	0.99(0.82–1.36)	0.947	0.97(0.90–1.05)	0.511	0.97(0.91–1.03)	0.314	0.831	0.904	12.5	0.823
Obesity due to excess calories	1.00(0.95–1.06)	0.938	1.01(0.99–1.02)	0.176	1.01(0.99–1.01)	0.283	0.177	0.907	23.6	0.167

**Figure 5:**
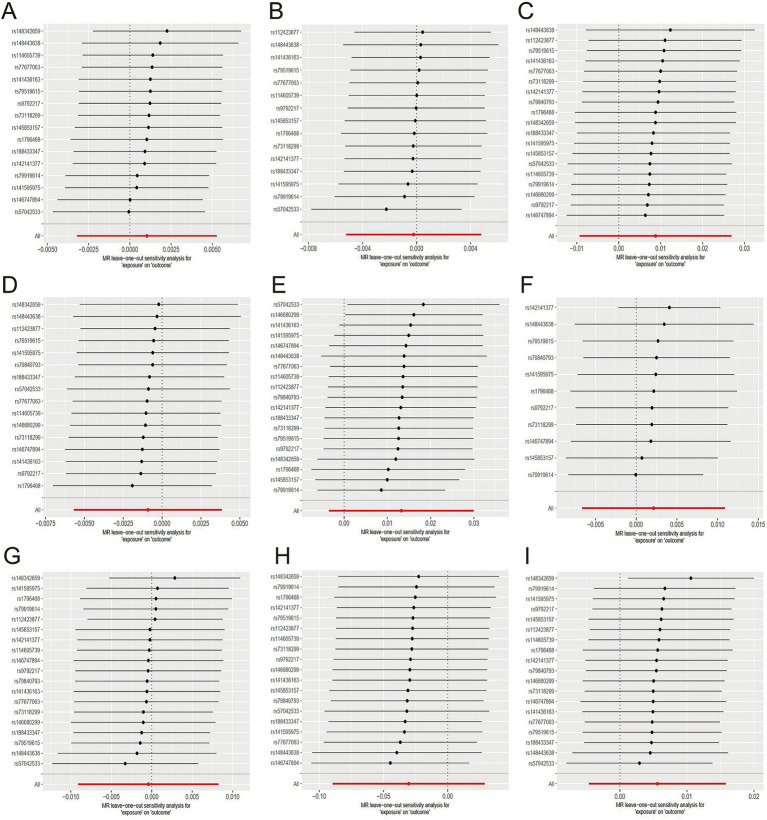
Sensitive analysis for MR analyses of the causal effect of herbal tea intake on hypertension, diabetes, and obesity. (A) hypertension; (B) essential hypertension; (C) secondary hypertension; (D) diabetes mellitus; (E) type 1 diabetes; (F) type 2 diabetes; (G) obesity; (H) obesity due to excess calories; (I) drug-induced obesity.

There was no causal association between herbal tea consumption and hypertension (OR = 1.00; 95% CI: 0.99–1.01), including both essential (OR = 1.00; 95% CI: 0.99–1.00) and secondary hypertension (OR = 1.01; 95% CI: 0.99–1.03). The leave-one-out sensitivity test confirmed the robustness of these findings (Figures 5A–C).

Similarly, there was no causal association between herbal tea consumption and diabetes (OR = 1.00; 95% CI: 0.99–1.00), encompassing type 1 diabetes (OR = 1.01; 95% CI: 0.99–1.03) and type 2 diabetes (OR = 1.00; 95% CI: 0.99–1.01). The results of sensitivity test were robust (Figures 5D–F).

There was no evidence suggesting that genetically predicted herbal tea consumption had a causal effect on obesity (OR = 1.00; 95% CI: 0.99–1.01), including drug-induced obesity (OR = 0.97; 95% CI: 0.91–1.03), and obesity due to excess calories (OR = 1.01; 95% CI: 0.99–1.01). The results of sensitivity test were robust (Figures 5G–I).

### Causal effect of hypertension, diabetes, and obesity on tea consumption

Supplementary Table S2 summarizes the causal association of hypertension, diabetes, and obesity with tea consumption.

No causal association existed between hypertension (OR = 1.00; 95% CI: 0.99–1.01), essential hypertension (OR = 1.00; 95% CI: 0.98–1.01), diabetes mellitus (OR = 0.99; 95% CI: 0.98–1.01), type 1 diabetes (OR = 1.00; 95% CI: 0.99–1.01), obesity (OR = 0.98; 95% CI: 0.95–1.01), and obesity due to excess calories (OR = 1.00; 95% CI: 0.99–1.01), and tea consumption. However, a slight causal association was identified between type 2 diabetes and tea consumption (OR = 0.99; 95% CI: 0.97–1.00). The sensitivity test results were consistent.

### Causal effect of hypertension, diabetes, and obesity on green tea intake

Supplementary Table S3 summarizes the causal association of hypertension, diabetes, and obesity with green tea intake.

No causal association existed between hypertension (OR = 0.93; 95% CI: 0.56–1.56), essential hypertension (OR = 0.95; 95% CI: 0.51–1.77), diabetes mellitus (OR = 0.92; 95% CI: 0.62–1.36), type 1 diabetes (OR = 0.85; 95% CI: 0.69–1.04), type 2 diabetes (OR = 1.06; 95% CI: 0.71–1.58), and obesity (OR = 0.57; 95% CI: 0.29–1.12) with green tea consumption. Interestingly, it was noted that individuals with obesity due to excess calories were less likely to consume green tea (OR = 0.35; 95% CI: 0.16–0.78). The sensitivity analysis results were consistent.

### Causal effect of hypertension, diabetes, and obesity on herbal tea intake

Supplementary Table S4 presents the causal association of hypertension, diabetes, and obesity with herbal tea intake.

No causal association was observed between hypertension (OR = 0.71; 95% CI: 0.42–1.20), essential hypertension (OR = 0.77; 95% CI: 0.41–1.46), diabetes mellitus (OR = 0.77; 95% CI: 0.52–1.14), type 1 diabetes (OR = 0.90; 95% CI: 0.73–1.10), type 2 diabetes (OR = 0.83; 95% CI: 0.57–1.21), obesity (OR = 1.08; 95% CI: 0.56–2.11), and obesity due to excess calories (OR = 0.86; 95% CI: 0.39–1.91), with herbal tea consumption. The sensitivity test results were reliable.

## Discussion

The bidirectional MR analysis revealed that tea consumption (including black and green teas) had a protective causal effect against hypertension. Further, our sensitivity analysis revealed that the relationship between tea consumption and the risks of type 2 diabetes and obesity due to excess caloric intake was modulated by SNPs, suggesting that these SNPs may possess unique biological functions. Conversely, reverse direction analysis indicated a negative association between type 2 diabetes and tea consumption, and a similar negative relationship between obesity from excess calories and green tea consumption.

Tea consumption’s effects on metabolic and cardiovascular diseases have been widely acknowledged, though the findings are mixed. Chieng et al. (26) reported cardiovascular benefits of high tea consumption akin to those associated with coffee, leading to improved survival rates in population studies. Zhang et al. (27) found that consuming 2 to 3 cups of tea daily was linked to a 32% lower risk of stroke and dementia. Niu et al. (28) noted that regular tea drinkers (≥3 times/week) had a 17% lower risk of developing dementia and a 14% decreased risk for hypertension. Another study by Zhang et al. (29) associated 3 to 4 cups of tea daily with the lowest all-cause mortality risk in individuals with hypertension. Grosso et al. (30) observed that high tea consumption correlated with lower metabolic syndrome risk and smaller waist circumferences, although not affecting diastolic blood pressure. Feng et al. (31) reported an association between habitual tea drinking, increased blood pressure, and higher hypertension risk among older Chinese adults. Our research corroborates and expands upon the findings of Grosso et al. and Feng et al., showing a protective effect of high tea consumption on hypertension. The results from Gao et al. MR study (32) support our findings, and Quan et al.’s prospective study (33) linked more than 1 cup of green tea daily to a reduced hypertension risk in premenopausal women. Our results primarily highlight the importance of the quantity of tea consumed daily, rather than the specific type of tea.

The recent increase in interest in dietary prevention of diabetes has highlighted polyphenolic compounds as promising natural agents for chemoprevention due to their health benefits. Although observational studies suggest a positive link between tea consumption and diabetes prevention, the evidence is not definitive and may be influenced by lifestyle and dietary factors (34). A meta-analysis by Yang et al. (35) indicated a 4.6% lower risk of type 2 diabetes (T2D) with an additional intake of two cups of tea daily. A multicenter, cross-sectional study reported a negative correlation between daily tea drinking and diabetes risk in elderly, obese, and female individuals (36). Another study (37) found that regular green tea consumption in Chinese adults was associated with a lower risk of T2D and reduced all-cause mortality among patients with diabetes. However, the effects of other tea types on these outcomes are less clear. Contrarily, Liu et al. (38) reported a contrary result that green tea drinkers had an increased T2D risk compared to non-drinkers. Another analysis by Yang et al. (39) showed no significant relationship between tea consumption and T2D risk, while Yang et al. (40) demonstrated a decreased diabetes risk with a daily intake of 3–4 cups of tea or more. Li et al. (41) supported these findings, noting a reduced T2D risk with high tea consumption (e.g., ≥4 cups/day). Our study found no causal link between tea intake and diabetes, but identified a significant influence of the SNP “rs57462170” on the association between tea consumption and T2D. Excluding this SNP revealed a protective effect of tea against T2D, suggesting that individual SNPs can significantly affect gene expression and disease outcomes.

Catechins have been considered to have a positive effect on weight loss. Hursel et al. (42) found that catechins significantly decreased body weight and helped maintain weight after a period of weight loss. A meta-analysis by Xu et al. (43) showed that green tea consumption significantly lowered low-density lipoprotein and total cholesterol levels. Another dose–response meta-analysis (44) revealed that green tea administration significantly reduced body weight and BMI. Experimental studies have shown that the anti-obesity mechanisms of tea mainly involve enhancing energy expenditure and lipid catabolism, suppressing nutrient digestion and absorption, and inhibiting lipid synthesis. These studies also highlight the modulation of adipocytes, the neuroendocrine system, and gut microbiota (45). However, Amozadeh et al. (46) found that green tea supplementation was not effective in weight loss. Mombaini et al. (47) conducted a randomized clinical trial and reported no significant difference in body weight and BMI between participants receiving green tea tablets and those receiving a placebo. Mechanistically, SNPs located in various regions can influence gene expression (48, 49). Specifically, nonsynonymous coding SNPs within the coding sequence directly alter the protein’s amino acid sequence (50). SNPs in introns mainly affect gene function by altering splice site activity. Regulatory regions, including promoters and enhancers, control gene expression. Changes in the expression levels of key genes can affect the body’s susceptibility to various diseases, ultimately influencing health outcomes. SNPs in introns primarily affect gene function by modifying splice site activity. The gene regulatory regions, which include promoter and enhancer regions, among others, control gene expression. Variations in SNPs within these areas can alter their ability to bind with regulatory factors, thereby impacting gene expression. Such changes can affect the expression levels of key genes, influencing the human body’s vulnerability to various diseases and leading to diverse health conditions.

In conducting a reverse direction analysis, it was noted that individuals with type 2 diabetes showed a tendency to consume less tea, whereas those with obesity, caused by excessive calorie intake, generally avoided green tea. This finding suggests a possible bidirectional relationship between tea consumption and both diabetes and obesity, highlighting the potential benefits of tea consumption in managing these conditions.

However, it’s crucial to note the limitations of our study. First, the SNPs related to tea consumption were extracted solely from the MRC IEU OpenGWAS dataset, while those linked to hypertension, diabetes, and obesity were taken from the FinnGen cohort, making it difficult to validate our MR findings with independent, high-quality datasets. Second, BMI was a significant confounder for hypertension, diabetes, and obesity. Using PhenoScanner V2, the results showed no overlap between SNPs related to BMI and the IVs used, which prevented further multivariate MR analysis. Other potential confounding factors also warrant consideration. Third, our analysis focused on the causal effects of tea consumption, indicating a one-standard deviation increase. The potential positive correlation could be missed if not comparing the highest to the lowest consumption categories. Third, although the tea consumption quantity variable includes both green and black tea, the UK Biobank database does not provide an independent dataset specifically for black tea. This limitation precludes us from separately assessing the effects of black tea on hypertension, diabetes, and obesity. Finally, the standard deviation of 2.84 for the mean tea consumption of 3.5 cups is relatively large and may be influenced by a few tea drinkers who consume more than 5 cups of tea per day. This suggests that the distribution of tea consumption requires careful interpretation. This study has also several strengths. First, we utilized a bidirectional Mendelian randomization approach to investigate the reciprocal causal associations between tea consumption and hypertension, diabetes, and obesity, thereby mitigating the risks of confounding factors and reverse causality. Second, we conducted subtype analyses for hypertension, diabetes, and obesity, which makes the results more targeted and specific.

## Conclusion

In summary, our bidirectional two-sample MR analyses suggest that consuming approximately 2.8 cups of tea daily (including black and green teas) may have a protective effect against hypertension. Moreover, increasing tea consumption quantity appeared to have a protective role against type 2 diabetes and obesity caused by excessive calorie intake, influenced by specific SNPs. Type 2 diabetes showed a negative association with tea consumption quantity, while obesity due to high calorie intake was negatively correlated with green tea consumption. In summary, the conclusion should be interpreted with caution.

## Data Availability

The original contributions presented in the study are included in the article/Supplementary material, further inquiries can be directed to the corresponding authors.

## Footnotes

^1^ https://finngen.gitbook.io/documentation/v/r5/data-download
