# Supplementary material for: Effect of tea consumption on the development of hypertension, diabetes, and obesity: a bidirectional two-sample Mendelian randomization analysis
Source: Front Nutr. 2024 Oct 31;11:1428445. doi: 10.3389/fnut.2024.1428445 (PMC11562749; doi:10.3389/fnut.2024.1428445)
Supplement: Supplementary file 1 [file Table_1.DOCX]

Supplementary Table 1. Genetic variants used as instrument variables

| **Exposure** | **rsID** | **EA** | **OA** | **EAF** | **Beta** | **SE** | **Pval** | **Sample size** | **R2** | **F statistic** |
| --- | --- | --- | --- | --- | --- | --- | --- | --- | --- | --- |
| Tea intake | rs11587444 | G | A | 0.393464 | 0.014033 | 0.002171 | 1.00E-10 | 447485 | 9.39897E-05 | 42.06274532 |
|  | rs11164870 | G | C | 0.604574 | -0.01196 | 0.002182 | 4.20E-08 | 447485 | 6.83968E-05 | 30.60852054 |
|  | rs56188862 | C | T | 0.387454 | -0.01576 | 0.002175 | 4.30E-13 | 447485 | 0.000117849 | 52.74151769 |
|  | rs1156588 | G | A | 0.210071 | -0.01545 | 0.002603 | 2.90E-09 | 447485 | 7.92622E-05 | 35.47128631 |
|  | rs57462170 | A | G | 0.108773 | 0.019151 | 0.003406 | 1.90E-08 | 447485 | 7.11049E-05 | 31.82050699 |
|  | rs2117137 | G | A | 0.405148 | 0.012995 | 0.002156 | 1.70E-09 | 447485 | 8.13939E-05 | 36.4253482 |
|  | rs1481012 | G | A | 0.112209 | -0.02624 | 0.003356 | 5.30E-15 | 447485 | 0.000137218 | 61.41126926 |
|  | rs34619 | A | G | 0.430905 | 0.011712 | 0.002138 | 4.30E-08 | 447485 | 6.72723E-05 | 30.10522903 |
|  | rs72797284 | G | A | 0.270797 | -0.01711 | 0.002384 | 7.00E-13 | 447485 | 0.000115681 | 51.77113142 |
|  | rs7757102 | G | A | 0.555426 | -0.0118 | 0.002133 | 3.10E-08 | 447485 | 6.881E-05 | 30.79340618 |
|  | rs2478875 | G | A | 0.208758 | 0.021894 | 0.002611 | 5.10E-17 | 447485 | 0.00015836 | 70.87448326 |
|  | rs149805207 | G | A | 0.008538 | -0.07193 | 0.012582 | 1.10E-08 | 447485 | 8.76046E-05 | 39.20501346 |
|  | rs4410790 | C | T | 0.631224 | 0.040551 | 0.002195 | 3.40E-76 | 447485 | 0.000765545 | 342.83082 |
|  | rs17685 | A | G | 0.277512 | 0.023066 | 0.002362 | 1.60E-22 | 447485 | 0.000213338 | 95.48548326 |
|  | rs141071726 | A | G | 0.026713 | 0.040732 | 0.006812 | 2.20E-09 | 447485 | 8.62715E-05 | 38.60834585 |
|  | rs9648476 | A | G | 0.622954 | 0.012501 | 0.002185 | 1.10E-08 | 447485 | 7.3416E-05 | 32.85482007 |
|  | rs713598 | G | C | 0.402254 | 0.013397 | 0.002157 | 5.20E-10 | 447485 | 8.63089E-05 | 38.62510767 |
|  | rs13282783 | T | C | 0.285899 | -0.01358 | 0.002354 | 7.90E-09 | 447485 | 7.53422E-05 | 33.71690476 |
|  | rs56348300 | G | C | 0.184619 | 0.015882 | 0.002732 | 6.10E-09 | 447485 | 7.5945E-05 | 33.98668146 |
|  | rs10764990 | A | G | 0.607155 | -0.01219 | 0.002169 | 1.90E-08 | 447485 | 7.08926E-05 | 31.72548679 |
|  | rs10752269 | A | G | 0.506082 | -0.01287 | 0.00212 | 1.30E-09 | 447485 | 8.28409E-05 | 37.07298506 |
|  | rs2351187 | A | G | 0.318935 | 0.012902 | 0.002282 | 1.60E-08 | 447485 | 7.23194E-05 | 32.36405728 |
|  | rs17245213 | A | G | 0.208046 | -0.01465 | 0.002609 | 2.00E-08 | 447485 | 7.07053E-05 | 31.64165303 |
|  | rs10741694 | C | T | 0.627915 | 0.015004 | 0.002194 | 7.90E-12 | 447485 | 0.000105189 | 47.07517964 |
|  | rs1453548 | A | T | 0.664929 | -0.01334 | 0.00225 | 3.00E-09 | 447485 | 7.93131E-05 | 35.49407889 |
|  | rs977474 | T | C | 0.833746 | 0.021781 | 0.002856 | 2.40E-14 | 447485 | 0.000131524 | 58.86228541 |
|  | rs2783129 | G | C | 0.484878 | -0.01173 | 0.002133 | 3.80E-08 | 447485 | 6.87699E-05 | 30.7754581 |
|  | rs17576658 | A | G | 0.247081 | -0.01348 | 0.002457 | 4.10E-08 | 447485 | 6.76199E-05 | 30.26081463 |
|  | rs6829 | T | C | 0.596155 | -0.01192 | 0.002165 | 3.70E-08 | 447485 | 6.83733E-05 | 30.59799642 |
|  | rs2645929 | G | A | 0.813066 | -0.01498 | 0.002717 | 3.50E-08 | 447485 | 6.82513E-05 | 30.54340147 |
|  | rs12591786 | T | C | 0.158804 | -0.01844 | 0.002942 | 3.70E-10 | 447485 | 9.0846E-05 | 40.65572889 |
|  | rs2472297 | T | C | 0.262049 | 0.053345 | 0.002401 | 2.30E-109 | 447485 | 0.001100607 | 493.0456637 |
|  | rs9937354 | A | G | 0.424074 | -0.01409 | 0.002143 | 4.90E-11 | 447485 | 9.70068E-05 | 43.41309613 |
|  | rs9302428 | G | C | 0.635799 | 0.012246 | 0.002201 | 2.60E-08 | 447485 | 6.94478E-05 | 31.07884742 |
|  | rs2279844 | A | G | 0.379343 | -0.01199 | 0.002183 | 4.00E-08 | 447485 | 6.76706E-05 | 30.28348709 |
|  | rs4808193 | C | T | 0.335324 | 0.015115 | 0.002247 | 1.70E-11 | 447485 | 0.000101839 | 45.57597407 |
|  | rs57631352 | G | A | 0.296859 | -0.0131 | 0.002321 | 1.70E-08 | 447485 | 7.16799E-05 | 32.07782668 |
|  | rs2273447 | T | A | 0.203788 | 0.017472 | 0.002634 | 3.30E-11 | 447485 | 9.90599E-05 | 44.33199452 |
|  | rs4817505 | C | T | 0.38998 | 0.015068 | 0.002175 | 4.20E-12 | 447485 | 0.000108026 | 48.34494675 |
|  | rs132904 | C | G | 0.778651 | 0.016601 | 0.002553 | 7.80E-11 | 447485 | 9.49955E-05 | 42.51292702 |
|  | rs9624470 | A | G | 0.580054 | 0.025207 | 0.002155 | 1.30E-31 | 447485 | 0.000309555 | 138.5634344 |
| Green tea intake | rs12144868 | C | T | 0.015146 | 3.07273 | 0.527463 | 5.70E-09 | 64949 | 0.281675194 | 25467.53042 |
|  | rs145313301 | C | G | 0.006149 | 4.99373 | 0.812513 | 7.90E-10 | 64949 | 0.304793627 | 28474.18045 |
|  | rs144954030 | C | T | 0.005665 | 4.88015 | 0.821066 | 2.80E-09 | 64949 | 0.268305131 | 23815.41009 |
|  | rs142811251 | C | G | 0.010296 | 3.45976 | 0.617814 | 2.10E-08 | 64949 | 0.24394718 | 20955.7283 |
|  | rs115952340 | A | G | 0.01334 | 3.00972 | 0.550266 | 4.50E-08 | 64949 | 0.238454507 | 20336.15197 |
|  | rs78547201 | G | C | 0.011607 | 3.27496 | 0.577735 | 1.40E-08 | 64949 | 0.246088682 | 21199.737 |
|  | rs116985617 | T | C | 0.008531 | 3.89273 | 0.674419 | 7.80E-09 | 64949 | 0.256340745 | 22387.35311 |
|  | rs79774709 | T | C | 0.005782 | 5.14056 | 0.853785 | 1.70E-09 | 64949 | 0.30381595 | 28342.98557 |
|  | rs189140232 | A | G | 0.010006 | 3.56083 | 0.625652 | 1.30E-08 | 64949 | 0.251203414 | 21788.17107 |
|  | rs11976995 | G | T | 0.030423 | 2.01942 | 0.348689 | 7.00E-09 | 64949 | 0.2405845 | 20575.3524 |
|  | rs116035596 | T | C | 0.006559 | 4.90357 | 0.742882 | 4.10E-11 | 64949 | 0.313353439 | 29638.77915 |
|  | rs79638269 | T | C | 0.016328 | 2.67635 | 0.472895 | 1.50E-08 | 64949 | 0.230090725 | 19409.69253 |
|  | rs644205 | A | G | 0.196485 | 0.848874 | 0.150756 | 1.80E-08 | 64949 | 0.227530619 | 19130.11896 |
|  | rs183788045 | T | G | 0.008749 | 3.54822 | 0.64978 | 4.70E-08 | 64949 | 0.218370078 | 18144.75249 |
|  | rs117251267 | C | T | 0.01159 | 3.55014 | 0.617762 | 9.10E-09 | 64949 | 0.288762985 | 26368.55106 |
|  | rs142373582 | C | T | 0.014186 | 3.24633 | 0.565762 | 9.60E-09 | 64949 | 0.294761164 | 27145.20578 |
|  | rs113898417 | G | C | 0.006957 | 3.97893 | 0.726631 | 4.40E-08 | 64949 | 0.218752312 | 18185.40598 |
|  | rs62059726 | A | G | 0.015194 | 2.8216 | 0.511814 | 3.50E-08 | 64949 | 0.238255918 | 20313.91839 |
|  | rs12958992 | G | A | 0.026945 | 2.16091 | 0.378903 | 1.20E-08 | 64949 | 0.244860612 | 21059.63803 |
|  | rs117077082 | G | A | 0.031795 | 1.92055 | 0.34217 | 2.00E-08 | 64949 | 0.227094901 | 19082.72119 |
|  | rs113322644 | A | C | 0.011694 | 3.20963 | 0.555077 | 7.40E-09 | 64949 | 0.238119224 | 20298.62115 |
| Herbal tea intake | rs142141377 | C | A | 0.006138 | 4.95948 | 0.797242 | 4.90E-10 | 64949 | 0.300092576 | 27846.70072 |
|  | rs145853157 | T | A | 0.008141 | 3.9211 | 0.672118 | 5.40E-09 | 64949 | 0.248298174 | 21452.94973 |
|  | rs141595975 | C | T | 0.009742 | 4.23585 | 0.669053 | 2.40E-10 | 64949 | 0.346184505 | 34388.36374 |
|  | rs79840793 | T | C | 0.011362 | 3.28994 | 0.571396 | 8.50E-09 | 64949 | 0.243163304 | 20866.75654 |
|  | rs114605739 | G | A | 0.010861 | 3.2706 | 0.584593 | 2.20E-08 | 64949 | 0.229832796 | 19381.44146 |
|  | rs1796468 | T | C | 0.007751 | 4.14799 | 0.707703 | 4.60E-09 | 64949 | 0.264657255 | 23375.07899 |
|  | rs148443638 | A | G | 0.009776 | 3.845 | 0.618252 | 5.00E-10 | 64949 | 0.286231433 | 26044.67855 |
|  | rs73118299 | G | A | 0.007719 | 4.30532 | 0.744377 | 7.30E-09 | 64949 | 0.283946543 | 25754.32874 |
|  | rs146680299 | T | C | 0.014203 | 2.86998 | 0.515989 | 2.70E-08 | 64949 | 0.230650986 | 19471.12339 |
|  | rs9792217 | T | C | 0.014049 | 2.93094 | 0.502233 | 5.40E-09 | 64949 | 0.237982266 | 20283.29993 |
|  | rs146747894 | A | G | 0.010457 | 3.42362 | 0.597023 | 9.80E-09 | 64949 | 0.242573237 | 20799.90412 |
|  | rs79519615 | C | T | 0.009933 | 3.45156 | 0.60022 | 8.90E-09 | 64949 | 0.234318118 | 19875.43285 |
|  | rs57042533 | T | G | 0.005412 | 4.5911 | 0.823545 | 2.50E-08 | 64949 | 0.226915678 | 19063.24075 |
|  | rs148342659 | T | C | 0.006752 | 4.24384 | 0.748342 | 1.40E-08 | 64949 | 0.241567293 | 20686.17402 |
|  | rs79919614 | T | A | 0.020412 | 2.53173 | 0.423009 | 2.20E-09 | 64949 | 0.256326665 | 22385.69967 |
|  | rs112423877 | A | T | 0.00698 | 4.05076 | 0.733715 | 3.40E-08 | 64949 | 0.227465973 | 19123.08332 |
|  | rs188433347 | G | T | 0.008704 | 3.63371 | 0.655449 | 3.00E-08 | 64949 | 0.227851955 | 19165.10836 |
|  | rs77677063 | T | C | 0.009871 | 3.53501 | 0.632623 | 2.30E-08 | 64949 | 0.244266676 | 20992.04477 |
|  | rs141436163 | T | G | 0.018642 | 2.49682 | 0.443009 | 1.70E-08 | 64949 | 0.228099554 | 19192.08854 |
| Hypertension | rs7545442 | T | C | 0.07246 | 0.098 | 0.0176 | 2.57E-08 | 218754 | 0.001290961 | 282.7653409 |
|  | rs1317181 | T | G | 0.2099 | 0.0701 | 0.0112 | 3.88E-10 | 218754 | 0.001629898 | 357.1256121 |
|  | rs143439093 | G | A | 0.07436 | -0.1373 | 0.0175 | 4.30E-15 | 218754 | 0.002595091 | 569.1583292 |
|  | rs6668768 | T | C | 0.2144 | 0.0668 | 0.0111 | 1.77E-09 | 218754 | 0.001503174 | 329.3172802 |
|  | rs3790604 | A | C | 0.1709 | 0.1321 | 0.0121 | 9.52E-28 | 218754 | 0.004945209 | 1087.15044 |
|  | rs34071855 | G | C | 0.415 | 0.1033 | 0.0093 | 1.15E-28 | 218754 | 0.005181251 | 1139.312001 |
|  | rs1374264 | C | A | 0.4146 | -0.0603 | 0.0092 | 5.59E-11 | 218754 | 0.001765008 | 386.781665 |
|  | rs10206833 | A | G | 0.8661 | -0.079 | 0.0134 | 3.73E-09 | 218754 | 0.001447547 | 317.112925 |
|  | rs1275984 | C | A | 0.549 | -0.0953 | 0.0092 | 3.82E-25 | 218754 | 0.004497433 | 988.2670855 |
|  | rs10185395 | T | G | 0.243 | 0.0587 | 0.0107 | 4.11E-08 | 218754 | 0.001267676 | 277.6586947 |
|  | rs4685218 | T | C | 0.09873 | 0.0984 | 0.0153 | 1.26E-10 | 218754 | 0.001723155 | 377.5941691 |
|  | rs2643826 | T | C | 0.4047 | 0.0627 | 0.0093 | 1.56E-11 | 218754 | 0.001894236 | 415.1543886 |
|  | rs16853076 | C | T | 0.06089 | -0.1096 | 0.0192 | 1.14E-08 | 218754 | 0.001373768 | 300.9280048 |
|  | rs12509595 | C | T | 0.3122 | 0.1157 | 0.0099 | 1.49E-31 | 218754 | 0.005748993 | 1264.875489 |
|  | rs3796585 | A | G | 0.3465 | -0.0751 | 0.0096 | 5.16E-15 | 218754 | 0.002554222 | 560.1720512 |
|  | rs149150643 | C | T | 0.04503 | -0.1328 | 0.0222 | 2.20E-09 | 218754 | 0.001516763 | 332.2990316 |
|  | rs13112725 | C | G | 0.8285 | 0.0697 | 0.0121 | 8.39E-09 | 218754 | 0.00138055 | 302.4156074 |
|  | rs10059884 | A | C | 0.5856 | 0.0651 | 0.0092 | 1.48E-12 | 218754 | 0.002056898 | 450.8779942 |
|  | rs4371736 | C | G | 0.3821 | -0.0527 | 0.0094 | 2.07E-08 | 218754 | 0.001311434 | 287.2555403 |
|  | rs188155432 | T | C | 0.02363 | 0.1977 | 0.0303 | 6.81E-11 | 218754 | 0.001803522 | 395.2368979 |
|  | rs6860901 | T | C | 0.2979 | 0.0682 | 0.01 | 9.10E-12 | 218754 | 0.001945666 | 426.4479869 |
|  | rs62426324 | T | C | 0.5009 | 0.0528 | 0.0091 | 6.55E-09 | 218754 | 0.001393915 | 305.3474284 |
|  | rs2032451 | T | G | 0.1098 | 0.0886 | 0.0146 | 1.29E-09 | 218754 | 0.001534572 | 336.2067048 |
|  | rs6918791 | G | C | 0.728 | 0.0574 | 0.0103 | 2.51E-08 | 218754 | 0.00130483 | 285.8071879 |
|  | rs62434119 | T | C | 0.07595 | -0.1345 | 0.0172 | 5.29E-15 | 218754 | 0.002539205 | 556.8702431 |
|  | rs2190994 | T | G | 0.276 | -0.06 | 0.0103 | 5.70E-09 | 218754 | 0.001438733 | 315.179136 |
|  | rs6961048 | G | C | 0.1355 | 0.0735 | 0.0133 | 3.27E-08 | 218754 | 0.001265636 | 277.2113489 |
|  | rs3918226 | T | C | 0.0699 | 0.1721 | 0.0179 | 6.94E-22 | 218754 | 0.003851222 | 845.7195738 |
|  | rs2392929 | G | T | 0.2979 | 0.062 | 0.01 | 5.65E-10 | 218754 | 0.001607988 | 352.3171513 |
|  | rs62455829 | G | A | 0.2782 | -0.0598 | 0.0103 | 6.40E-09 | 218754 | 0.001436172 | 314.6172779 |
|  | rs10217559 | T | C | 0.6936 | 0.0584 | 0.0099 | 3.66E-09 | 218754 | 0.001449618 | 317.5671602 |
|  | rs10757274 | G | A | 0.432 | 0.0507 | 0.0092 | 3.57E-08 | 218754 | 0.001261473 | 276.2983083 |
|  | rs72779268 | A | C | 0.06436 | -0.1222 | 0.0187 | 6.37E-11 | 218754 | 0.001798445 | 394.1223046 |
|  | rs2274224 | C | G | 0.3456 | -0.0751 | 0.0096 | 5.16E-15 | 218754 | 0.002551097 | 559.4847705 |
|  | rs1888693 | A | G | 0.386 | 0.06 | 0.0094 | 1.74E-10 | 218754 | 0.001706429 | 373.9227855 |
|  | rs10786736 | C | G | 0.09173 | -0.1089 | 0.0158 | 5.49E-12 | 218754 | 0.001976115 | 433.1349369 |
|  | rs2782981 | C | T | 0.6546 | 0.0799 | 0.0096 | 8.58E-17 | 218754 | 0.002886835 | 633.3291394 |
|  | rs7483477 | G | T | 0.2348 | 0.0759 | 0.0109 | 3.32E-12 | 218754 | 0.002070077 | 453.7729347 |
|  | rs557675 | G | T | 0.3969 | -0.0613 | 0.0093 | 4.36E-11 | 218754 | 0.001798959 | 394.2351889 |
|  | rs7123467 | G | C | 0.202 | 0.0682 | 0.0114 | 2.20E-09 | 218754 | 0.001499523 | 328.5161778 |
|  | rs604723 | C | T | 0.741 | 0.0632 | 0.0104 | 1.23E-09 | 218754 | 0.001533141 | 335.8926512 |
|  | rs7134677 | T | C | 0.3819 | -0.0748 | 0.0094 | 1.76E-15 | 218754 | 0.002641445 | 579.3517305 |
|  | rs7310615 | G | C | 0.5863 | -0.0816 | 0.0093 | 1.72E-18 | 218754 | 0.003230098 | 708.8801982 |
|  | rs1358350 | A | T | 0.7541 | 0.0663 | 0.0106 | 3.98E-10 | 218754 | 0.001630214 | 357.1947975 |
|  | rs12828438 | G | A | 0.5354 | -0.0606 | 0.0092 | 4.49E-11 | 218754 | 0.001826976 | 400.3861258 |
|  | rs35427 | G | T | 0.3644 | -0.0592 | 0.0096 | 6.97E-10 | 218754 | 0.001623438 | 355.7077449 |
|  | rs303949 | C | A | 0.09934 | 0.0849 | 0.0153 | 2.87E-08 | 218754 | 0.001289824 | 282.5159452 |
|  | rs629929 | T | G | 0.5795 | 0.0595 | 0.0093 | 1.58E-10 | 218754 | 0.001725374 | 378.0814481 |
|  | rs2627313 | T | C | 0.4006 | 0.054 | 0.0093 | 6.38E-09 | 218754 | 0.001400378 | 306.7650184 |
|  | rs4932373 | C | A | 0.2674 | 0.0837 | 0.0103 | 4.43E-16 | 218754 | 0.002744791 | 602.0810352 |
|  | rs11636952 | C | T | 0.6071 | -0.055 | 0.0094 | 4.88E-09 | 218754 | 0.001443104 | 316.1381107 |
|  | rs28567725 | C | T | 0.4136 | 0.0617 | 0.0093 | 3.26E-11 | 218754 | 0.001846608 | 404.6966049 |
|  | rs35619711 | G | C | 0.7217 | 0.0614 | 0.0102 | 1.75E-09 | 218754 | 0.001514386 | 331.7774539 |
|  | rs79873333 | T | C | 0.04758 | -0.1364 | 0.0215 | 2.24E-10 | 218754 | 0.00168621 | 369.4848561 |
|  | rs117515309 | A | C | 0.06182 | -0.1088 | 0.019 | 1.03E-08 | 218754 | 0.001373102 | 300.7819223 |
|  | rs9899012 | A | G | 0.08387 | -0.1068 | 0.0166 | 1.25E-10 | 218754 | 0.001752816 | 384.1052008 |
|  | rs145153053 | G | A | 0.1963 | 0.0707 | 0.0115 | 7.86E-10 | 218754 | 0.001577187 | 345.5577422 |
|  | rs167479 | T | G | 0.4249 | -0.0833 | 0.0093 | 3.34E-19 | 218754 | 0.003391174 | 744.3503564 |
|  | rs189498068 | G | A | 0.01165 | -0.2554 | 0.0436 | 4.69E-09 | 218754 | 0.001502133 | 329.0889989 |
|  | rs78412528 | A | G | 0.1674 | 0.1406 | 0.0122 | 9.92E-31 | 218754 | 0.005510519 | 1212.116424 |
| Essential hypertension | rs880315 | C | T | 0.4127 | 0.0955 | 0.01 | 1.30E-21 | 205694 | 0.004421109 | 913.4250753 |
|  | rs6668768 | T | C | 0.2141 | 0.0706 | 0.012 | 4.02E-09 | 205694 | 0.001677349 | 345.5968932 |
|  | rs10776752 | T | G | 0.1691 | 0.1277 | 0.0131 | 1.88E-22 | 205694 | 0.004582518 | 946.9265508 |
|  | rs1317181 | T | G | 0.2096 | 0.0731 | 0.0121 | 1.53E-09 | 205694 | 0.001770529 | 364.8295208 |
|  | rs143439093 | G | A | 0.07434 | -0.1482 | 0.0189 | 4.46E-15 | 205694 | 0.003022738 | 623.638049 |
|  | rs34924059 | T | G | 0.1554 | 0.0746 | 0.0136 | 4.13E-08 | 205694 | 0.001460864 | 300.9276212 |
|  | rs1275984 | C | A | 0.5499 | -0.0901 | 0.0099 | 8.95E-20 | 205694 | 0.004018577 | 829.9242855 |
|  | rs2704368 | G | A | 0.8376 | -0.0858 | 0.0133 | 1.11E-10 | 205694 | 0.002002752 | 412.7768404 |
|  | rs3906954 | T | C | 0.092 | 0.0929 | 0.017 | 4.64E-08 | 205694 | 0.0014419 | 297.0155326 |
|  | rs16998073 | T | A | 0.3113 | 0.1076 | 0.0106 | 3.28E-24 | 205694 | 0.004964365 | 1026.224807 |
|  | rs149150643 | C | T | 0.04522 | -0.1374 | 0.0238 | 7.78E-09 | 205694 | 0.001630187 | 335.8638732 |
|  | rs3796582 | C | T | 0.3279 | -0.0641 | 0.0104 | 7.12E-10 | 205694 | 0.001811012 | 373.1865589 |
|  | rs12656497 | C | T | 0.5852 | 0.0646 | 0.0099 | 6.79E-11 | 205694 | 0.002025994 | 417.57671 |
|  | rs6860901 | T | C | 0.2971 | 0.0636 | 0.0107 | 2.78E-09 | 205694 | 0.001689431 | 348.0904866 |
|  | rs62434119 | T | C | 0.0761 | -0.1384 | 0.0185 | 7.37E-14 | 205694 | 0.002693468 | 555.5210719 |
|  | rs2032451 | T | G | 0.1097 | 0.0908 | 0.0157 | 7.32E-09 | 205694 | 0.001610441 | 331.7890614 |
|  | rs3918226 | T | C | 0.06939 | 0.1566 | 0.0194 | 6.91E-16 | 205694 | 0.003167219 | 653.5415491 |
|  | rs17477177 | C | T | 0.2975 | 0.0684 | 0.0107 | 1.63E-10 | 205694 | 0.00195558 | 403.0352462 |
|  | rs72779268 | A | C | 0.0646 | -0.1197 | 0.0201 | 2.60E-09 | 205694 | 0.001731602 | 356.794588 |
|  | rs10786736 | C | G | 0.09206 | -0.1093 | 0.0169 | 9.97E-11 | 205694 | 0.001997094 | 411.6082158 |
|  | rs2484294 | A | G | 0.7473 | 0.0854 | 0.0113 | 4.11E-14 | 205694 | 0.00275452 | 568.1477429 |
|  | rs2274224 | C | G | 0.3465 | -0.0638 | 0.0103 | 5.86E-10 | 205694 | 0.001843403 | 379.873417 |
|  | rs7483477 | G | T | 0.2345 | 0.0738 | 0.0117 | 2.83E-10 | 205694 | 0.001955378 | 402.9936506 |
|  | rs557675 | G | T | 0.3977 | -0.0551 | 0.01 | 3.59E-08 | 205694 | 0.00145446 | 299.6064592 |
|  | rs12828438 | G | A | 0.5356 | -0.0642 | 0.0099 | 8.88E-11 | 205694 | 0.002050373 | 422.611793 |
|  | rs7310615 | G | C | 0.5868 | -0.0789 | 0.01 | 3.02E-15 | 205694 | 0.003018801 | 622.8233016 |
|  | rs7134677 | T | C | 0.3829 | -0.0632 | 0.0101 | 3.91E-10 | 205694 | 0.001887579 | 388.9940922 |
|  | rs506409 | T | C | 0.5794 | 0.061 | 0.01 | 1.06E-09 | 205694 | 0.001813583 | 373.717272 |
|  | rs303949 | C | A | 0.09928 | 0.0981 | 0.0164 | 2.21E-09 | 205694 | 0.001721153 | 354.6378762 |
|  | rs1894400 | T | C | 0.2708 | 0.0811 | 0.0111 | 2.75E-13 | 205694 | 0.002597569 | 535.6905714 |
|  | rs9930501 | G | A | 0.4243 | 0.0616 | 0.0099 | 4.90E-10 | 205694 | 0.001853791 | 382.0180825 |
|  | rs149441293 | C | G | 0.04467 | 0.1429 | 0.0239 | 2.24E-09 | 205694 | 0.001742865 | 359.1193446 |
|  | rs8068318 | T | C | 0.7215 | 0.0603 | 0.0109 | 3.16E-08 | 205694 | 0.001461255 | 301.0084138 |
|  | rs145153053 | G | A | 0.196 | 0.0689 | 0.0124 | 2.75E-08 | 205694 | 0.001496169 | 308.2110641 |
|  | rs78008010 | C | A | 0.04681 | -0.1486 | 0.0233 | 1.80E-10 | 205694 | 0.001970542 | 406.1250464 |
|  | rs167479 | T | G | 0.4256 | -0.0822 | 0.01 | 2.04E-16 | 205694 | 0.003303617 | 681.7799104 |
|  | rs78412528 | A | G | 0.1663 | 0.1231 | 0.0132 | 1.10E-20 | 205694 | 0.004201924 | 867.9491261 |
| Diabetes mellitus | rs6679677 | A | C | 0.1472 | 0.0138 | 0.1116 | 6.12E-16 | 218792 | 4.78127E-05 | 10.46143844 |
|  | rs2943656 | G | A | 0.6175 | 0.0101 | 0.0694 | 6.36E-12 | 218792 | 4.81882E-05 | 10.54361505 |
|  | rs10184004 | T | C | 0.3577 | 0.0103 | -0.0582 | 1.60E-08 | 218792 | 4.87485E-05 | 10.66620551 |
|  | rs62137406 | T | C | 0.04899 | 0.0226 | 0.1333 | 3.67E-09 | 218792 | 4.75926E-05 | 10.41327973 |
|  | rs112694524 | A | G | 0.03339 | 0.0278 | -0.1717 | 6.56E-10 | 218792 | 4.9887E-05 | 10.91531881 |
|  | rs62146643 | T | C | 0.02859 | 0.0294 | 0.1626 | 3.19E-08 | 218792 | 4.80111E-05 | 10.50484628 |
|  | rs17039732 | A | T | 0.04772 | 0.023 | 0.1563 | 1.08E-11 | 218792 | 4.80785E-05 | 10.5195973 |
|  | rs11720108 | T | C | 0.176 | 0.0129 | -0.0846 | 5.45E-11 | 218792 | 4.82669E-05 | 10.56082209 |
|  | rs6780171 | A | T | 0.3065 | 0.0106 | 0.0845 | 1.57E-15 | 218792 | 4.7766E-05 | 10.45121745 |
|  | rs11709077 | A | G | 0.1707 | 0.0131 | -0.094 | 7.20E-13 | 218792 | 4.85867E-05 | 10.63080968 |
|  | rs6550758 | C | A | 0.8216 | 0.0128 | -0.0754 | 3.85E-09 | 218792 | 4.80292E-05 | 10.50881008 |
|  | rs3887925 | T | C | 0.463 | 0.0099 | 0.0547 | 3.29E-08 | 218792 | 4.87366E-05 | 10.66361106 |
|  | rs1046317 | C | T | 0.6107 | 0.0101 | 0.0762 | 4.54E-14 | 218792 | 4.85048E-05 | 10.61288849 |
|  | rs10938397 | G | A | 0.4736 | 0.0098 | 0.0643 | 5.34E-11 | 218792 | 4.78861E-05 | 10.47750766 |
|  | rs76177300 | A | G | 0.05783 | 0.021 | 0.1269 | 1.51E-09 | 218792 | 4.80564E-05 | 10.51476058 |
|  | rs3131640 | T | A | 0.5721 | 0.0108 | 0.0992 | 4.11E-20 | 218792 | 5.71073E-05 | 12.495223 |
|  | rs62398467 | T | C | 0.2543 | 0.0113 | 0.0913 | 6.50E-16 | 218792 | 4.84281E-05 | 10.59609606 |
|  | rs3998159 | C | A | 0.1211 | 0.0152 | 0.3936 | 7.63E-148 | 218792 | 4.91814E-05 | 10.76092507 |
|  | rs1815311 | G | A | 0.4053 | 0.01 | 0.0553 | 3.20E-08 | 218792 | 4.82064E-05 | 10.54758278 |
|  | rs9348441 | A | T | 0.3289 | 0.0104 | 0.1127 | 2.31E-27 | 218792 | 4.77472E-05 | 10.44710577 |
|  | rs878521 | A | G | 0.2071 | 0.0121 | 0.0731 | 1.53E-09 | 218792 | 4.80839E-05 | 10.52077189 |
|  | rs77655131 | T | C | 0.1836 | 0.0127 | 0.0906 | 9.76E-13 | 218792 | 4.83519E-05 | 10.57941316 |
|  | rs6977081 | T | G | 0.3593 | 0.0102 | 0.0654 | 1.44E-10 | 218792 | 4.79007E-05 | 10.48070633 |
|  | rs11558471 | G | A | 0.3784 | 0.0101 | -0.0685 | 1.18E-11 | 218792 | 4.79882E-05 | 10.49985222 |
|  | rs10974438 | C | A | 0.376 | 0.0101 | 0.0608 | 1.75E-09 | 218792 | 4.7868E-05 | 10.47353855 |
|  | rs28505901 | G | A | 0.6986 | 0.0107 | 0.0883 | 1.55E-16 | 218792 | 4.82136E-05 | 10.54915778 |
|  | rs7018475 | G | T | 0.2791 | 0.011 | 0.1034 | 5.46E-21 | 218792 | 4.86912E-05 | 10.65366026 |
|  | rs10882099 | C | T | 0.4766 | 0.0098 | -0.0817 | 7.64E-17 | 218792 | 4.79148E-05 | 10.48378682 |
|  | rs114322470 | G | T | 0.02355 | 0.0326 | -0.1957 | 1.94E-09 | 218792 | 4.88772E-05 | 10.69436033 |
|  | rs7903146 | T | C | 0.1996 | 0.0124 | 0.2675 | 3.25E-103 | 218792 | 4.91293E-05 | 10.74953773 |
|  | rs11257658 | A | G | 0.2648 | 0.0112 | 0.0715 | 1.73E-10 | 218792 | 4.88416E-05 | 10.6865667 |
|  | rs2237897 | T | C | 0.08141 | 0.0182 | -0.1591 | 2.30E-18 | 218792 | 4.95419E-05 | 10.83979887 |
|  | rs10830963 | G | C | 0.3567 | 0.0102 | 0.1063 | 1.98E-25 | 218792 | 4.77471E-05 | 10.44708685 |
|  | rs5215 | T | C | 0.5284 | 0.0098 | -0.0538 | 4.02E-08 | 218792 | 4.78651E-05 | 10.47290125 |
|  | rs7109575 | A | G | 0.2373 | 0.0116 | -0.0874 | 4.90E-14 | 218792 | 4.87077E-05 | 10.65727276 |
|  | rs74862545 | T | C | 0.01951 | 0.0366 | -0.2256 | 7.10E-10 | 218792 | 5.12499E-05 | 11.21352953 |
|  | rs76895963 | G | T | 0.03114 | 0.0306 | -0.4402 | 6.37E-47 | 218792 | 5.65005E-05 | 12.36244834 |
|  | rs10466811 | A | G | 0.2372 | 0.0116 | -0.0638 | 3.80E-08 | 218792 | 4.86935E-05 | 10.65417827 |
|  | rs2583921 | C | A | 0.0563 | 0.0214 | 0.137 | 1.53E-10 | 218792 | 4.86631E-05 | 10.64752075 |
|  | rs112108223 | A | G | 0.02237 | 0.0343 | -0.3232 | 4.39E-21 | 218792 | 5.14587E-05 | 11.25922653 |
|  | rs78470967 | A | T | 0.03942 | 0.0258 | -0.2078 | 8.00E-16 | 218792 | 5.04103E-05 | 11.02983281 |
|  | rs56348580 | C | G | 0.2827 | 0.011 | -0.0704 | 1.55E-10 | 218792 | 4.90729E-05 | 10.73719366 |
|  | rs7998259 | A | G | 0.3894 | 0.0101 | -0.069 | 8.39E-12 | 218792 | 4.85094E-05 | 10.61387641 |
|  | rs34337125 | A | G | 0.4142 | 0.01 | -0.0568 | 1.35E-08 | 218792 | 4.85277E-05 | 10.61788462 |
|  | rs9933509 | C | T | 0.4128 | 0.0099 | 0.1019 | 7.58E-25 | 218792 | 4.75145E-05 | 10.39619039 |
|  | rs55993634 | G | C | 0.08746 | 0.0174 | -0.1001 | 8.77E-09 | 218792 | 4.8327E-05 | 10.57397629 |
|  | rs3110641 | G | A | 0.7867 | 0.0121 | 0.0662 | 4.47E-08 | 218792 | 4.91361E-05 | 10.75101704 |
|  | rs11263763 | A | G | 0.6458 | 0.0103 | -0.0577 | 2.12E-08 | 218792 | 4.85346E-05 | 10.61939046 |
|  | rs11665052 | G | A | 0.1948 | 0.0124 | 0.0752 | 1.32E-09 | 218792 | 4.82354E-05 | 10.55393711 |
|  | rs8100204 | A | G | 0.1599 | 0.0135 | 0.0891 | 4.11E-11 | 218792 | 4.8964E-05 | 10.71336039 |
|  | rs2303700 | C | T | 0.674 | 0.0105 | -0.06 | 1.10E-08 | 218792 | 4.84491E-05 | 10.60070137 |
|  | rs429358 | C | T | 0.1826 | 0.0129 | -0.0757 | 4.41E-09 | 218792 | 4.96758E-05 | 10.86910704 |
|  | rs4812812 | T | C | 0.1972 | 0.0123 | 0.0776 | 2.81E-10 | 218792 | 4.79021E-05 | 10.48100107 |
|  | rs45551238 | T | C | 0.0499 | 0.023 | -0.21 | 6.82E-20 | 218792 | 5.01598E-05 | 10.97500646 |
|  | rs8353 | T | G | 0.3008 | 0.0107 | -0.0649 | 1.32E-09 | 218792 | 4.81589E-05 | 10.53719911 |
| DM1 | rs6679677 | A | C | 0.1465 | 0.0434 | 0.5854 | 1.83E-41 | 186323 | 0.000471032 | 87.80455094 |
|  | rs2260051 | T | A | 0.4927 | 0.0322 | 0.5577 | 3.33E-67 | 186323 | 0.000518309 | 96.62202327 |
|  | rs707958 | A | C | 0.5326 | 0.0313 | -0.2818 | 2.19E-19 | 186323 | 0.000487763 | 90.92477429 |
|  | rs1233386 | T | C | 0.1523 | 0.042 | 0.4512 | 6.40E-27 | 186323 | 0.000455481 | 84.90442546 |
|  | rs9260231 | T | G | 0.072 | 0.065 | -0.5773 | 6.59E-19 | 186323 | 0.000564595 | 105.2553689 |
|  | rs9268833 | T | C | 0.2292 | 0.0378 | 1.1716 | 6.35E-211 | 186323 | 0.000504859 | 94.11330692 |
|  | rs10947458 | T | C | 0.279 | 0.0354 | -0.2524 | 1.00E-12 | 186323 | 0.000504169 | 93.98462374 |
|  | rs1794269 | T | C | 0.5114 | 0.0314 | 1.1768 | 2.13E-307 | 186323 | 0.000492724 | 91.85003465 |
|  | rs689 | T | A | 0.7913 | 0.041 | 0.5794 | 2.42E-45 | 186323 | 0.000555215 | 103.5057137 |
| DM2 | rs2943656 | G | A | 0.6172 | 0.0764 | 0.0111 | 5.87E-12 | 212351 | 0.002758129 | 587.3057794 |
|  | rs13389219 | T | C | 0.3476 | -0.0706 | 0.0114 | 5.90E-10 | 212351 | 0.002260649 | 481.1342092 |
|  | rs112694524 | A | G | 0.03348 | -0.1807 | 0.0306 | 3.52E-09 | 212351 | 0.00211321 | 449.688252 |
|  | rs62137406 | T | C | 0.04888 | 0.1402 | 0.0249 | 1.80E-08 | 212351 | 0.001827648 | 388.8098135 |
|  | rs11712037 | G | C | 0.1708 | -0.1091 | 0.0144 | 3.55E-14 | 212351 | 0.003371527 | 718.3623894 |
|  | rs71330995 | A | G | 0.1879 | -0.0928 | 0.0139 | 2.45E-11 | 212351 | 0.002628223 | 559.5712388 |
|  | rs6786846 | A | G | 0.681 | 0.0733 | 0.0116 | 2.63E-10 | 212351 | 0.002334403 | 496.8679265 |
|  | rs3887925 | T | C | 0.4629 | 0.0609 | 0.0109 | 2.31E-08 | 212351 | 0.001844195 | 392.3365759 |
|  | rs6780171 | A | T | 0.3063 | 0.094 | 0.0117 | 9.42E-16 | 212351 | 0.003754952 | 800.365645 |
|  | rs1046317 | C | T | 0.6105 | 0.0842 | 0.0111 | 3.31E-14 | 212351 | 0.003371687 | 718.3966666 |
|  | rs745805 | T | A | 0.1503 | -0.083 | 0.0152 | 4.75E-08 | 212351 | 0.001759587 | 374.3051922 |
|  | rs10938397 | G | A | 0.4736 | 0.0742 | 0.0108 | 6.40E-12 | 212351 | 0.002745146 | 584.5335481 |
|  | rs76177300 | A | G | 0.05776 | 0.1386 | 0.0232 | 2.31E-09 | 212351 | 0.002090957 | 444.9430627 |
|  | rs9505086 | C | T | 0.4439 | 0.064 | 0.011 | 5.95E-09 | 212351 | 0.002022218 | 430.2861143 |
|  | rs7451008 | C | T | 0.3292 | 0.128 | 0.0115 | 8.92E-29 | 212351 | 0.007236071 | 1547.772216 |
|  | rs2781655 | T | C | 0.2218 | 0.0766 | 0.013 | 3.81E-09 | 212351 | 0.002025538 | 430.993872 |
|  | rs498475 | A | G | 0.6449 | -0.0656 | 0.0113 | 6.42E-09 | 212351 | 0.001970973 | 419.3607413 |
|  | rs878521 | A | G | 0.2072 | 0.0894 | 0.0133 | 1.79E-11 | 212351 | 0.002625781 | 559.0498145 |
|  | rs77655131 | T | C | 0.1834 | 0.0996 | 0.014 | 1.12E-12 | 212351 | 0.002971374 | 632.8488163 |
|  | rs62492368 | A | G | 0.3401 | 0.0768 | 0.0114 | 1.62E-11 | 212351 | 0.002647507 | 563.6879342 |
|  | rs11558471 | G | A | 0.3785 | -0.0814 | 0.0112 | 3.65E-13 | 212351 | 0.003117352 | 664.0365824 |
|  | rs7018475 | G | T | 0.2788 | 0.1144 | 0.0121 | 3.24E-21 | 212351 | 0.005262966 | 1123.498417 |
|  | rs28642213 | G | A | 0.6971 | 0.1004 | 0.0118 | 1.76E-17 | 212351 | 0.004256884 | 907.8094195 |
|  | rs11257658 | A | G | 0.2647 | 0.0854 | 0.0123 | 3.84E-12 | 212351 | 0.002838992 | 604.573597 |
|  | rs10882099 | C | T | 0.4772 | -0.0788 | 0.0108 | 2.96E-13 | 212351 | 0.003098264 | 659.9580224 |
|  | rs144155527 | T | C | 0.0236 | -0.2166 | 0.036 | 1.78E-09 | 212351 | 0.002162154 | 460.1261569 |
|  | rs34872471 | C | T | 0.2012 | 0.3047 | 0.0137 | 1.38E-109 | 212351 | 0.029842894 | 6532.043794 |
|  | rs182788819 | T | C | 0.03871 | 0.1576 | 0.0281 | 2.04E-08 | 212351 | 0.001848502 | 393.2545673 |
|  | rs73541184 | A | G | 0.3086 | -0.0863 | 0.0117 | 1.63E-13 | 212351 | 0.003178168 | 677.0325721 |
|  | rs10743152 | C | T | 0.6623 | -0.0657 | 0.0114 | 8.26E-09 | 212351 | 0.001930841 | 410.8053916 |
|  | rs2237897 | T | C | 0.08138 | -0.1982 | 0.0201 | 6.16E-23 | 212351 | 0.005873418 | 1254.583038 |
|  | rs5215 | T | C | 0.5286 | -0.0619 | 0.0108 | 9.96E-09 | 212351 | 0.001909537 | 406.2640044 |
|  | rs10830963 | G | C | 0.3567 | 0.1316 | 0.0113 | 2.40E-31 | 212351 | 0.007948011 | 1701.273835 |
|  | rs112108223 | A | G | 0.02242 | -0.3709 | 0.038 | 1.66E-22 | 212351 | 0.006030198 | 1288.275087 |
|  | rs78470967 | A | T | 0.03949 | -0.2426 | 0.0285 | 1.71E-17 | 212351 | 0.004464786 | 952.3447822 |
|  | rs74862545 | T | C | 0.01953 | -0.258 | 0.0405 | 1.89E-10 | 212351 | 0.002549212 | 542.7061001 |
|  | rs76895963 | G | T | 0.03124 | -0.5069 | 0.0339 | 1.49E-50 | 212351 | 0.015552557 | 3354.744787 |
|  | rs73113806 | T | C | 0.02331 | 0.2548 | 0.0359 | 1.27E-12 | 212351 | 0.002956159 | 629.5987004 |
|  | rs1798085 | C | T | 0.5575 | -0.0598 | 0.0109 | 4.11E-08 | 212351 | 0.001764373 | 375.3251484 |
|  | rs56348580 | C | G | 0.2829 | -0.0785 | 0.0121 | 8.72E-11 | 212351 | 0.002500242 | 532.2545713 |
|  | rs7998259 | A | G | 0.3896 | -0.0769 | 0.0112 | 6.60E-12 | 212351 | 0.002812653 | 598.9486761 |
|  | rs28553330 | C | T | 0.06049 | -0.1283 | 0.0229 | 2.11E-08 | 212351 | 0.001870976 | 398.0446911 |
|  | rs12449219 | G | C | 0.05833 | 0.1307 | 0.0232 | 1.76E-08 | 212351 | 0.001876601 | 399.2435101 |
|  | rs9940128 | A | G | 0.4287 | 0.1176 | 0.0109 | 3.88E-27 | 212351 | 0.006774268 | 1448.320254 |
|  | rs55993634 | G | C | 0.08714 | -0.1646 | 0.0194 | 2.17E-17 | 212351 | 0.004310339 | 919.2584123 |
|  | rs7224685 | T | G | 0.3016 | 0.0644 | 0.0117 | 3.71E-08 | 212351 | 0.001747179 | 371.6609837 |
|  | rs11263763 | A | G | 0.646 | -0.0665 | 0.0113 | 3.98E-09 | 212351 | 0.002022596 | 430.3666188 |
|  | rs12967878 | C | T | 0.181 | 0.077 | 0.014 | 3.80E-08 | 212351 | 0.001757818 | 373.9282054 |
|  | rs2303700 | C | T | 0.6741 | -0.0675 | 0.0116 | 5.92E-09 | 212351 | 0.002001918 | 425.9579638 |
|  | rs8100204 | A | G | 0.1597 | 0.0975 | 0.0149 | 6.00E-11 | 212351 | 0.0025514 | 543.1730347 |
|  | rs429358 | C | T | 0.1827 | -0.0813 | 0.0142 | 1.03E-08 | 212351 | 0.001973927 | 419.9904992 |
|  | rs7507893 | A | G | 0.4965 | -0.0611 | 0.0109 | 2.08E-08 | 212351 | 0.001866514 | 397.0934633 |
|  | rs45551238 | T | C | 0.05006 | -0.2295 | 0.0253 | 1.18E-19 | 212351 | 0.005009362 | 1069.088411 |
|  | rs77735929 | A | T | 0.04278 | 0.1758 | 0.0269 | 6.35E-11 | 212351 | 0.002531164 | 538.8540679 |
|  | rs117657619 | A | G | 0.0476 | -0.1414 | 0.0258 | 4.24E-08 | 212351 | 0.001812822 | 385.6500458 |
|  | rs6017317 | G | T | 0.2185 | 0.0768 | 0.0131 | 4.56E-09 | 212351 | 0.00201434 | 428.6065254 |
|  | rs8353 | T | G | 0.3009 | -0.0744 | 0.0118 | 2.88E-10 | 212351 | 0.002328828 | 495.6785816 |
| Obesity | rs12623218 | A | T | 0.8373 | 0.1511 | 0.0227 | 2.81E-11 | 218735 | 0.006220533 | 1369.152609 |
|  | rs734597 | A | G | 0.2012 | 0.1271 | 0.0206 | 6.83E-10 | 218735 | 0.005192627 | 1141.727452 |
|  | rs71245092 | A | G | 0.1865 | 0.1327 | 0.0214 | 5.61E-10 | 218735 | 0.005343284 | 1175.031001 |
|  | rs11030104 | G | A | 0.1662 | -0.1592 | 0.0225 | 1.49E-12 | 218735 | 0.007024397 | 1547.336478 |
|  | rs1558902 | A | T | 0.4194 | 0.2279 | 0.0168 | 6.42E-42 | 218735 | 0.025294384 | 5676.294828 |
|  | rs117280037 | C | T | 0.01587 | 0.3779 | 0.0678 | 2.49E-08 | 218735 | 0.004460804 | 980.0971431 |
|  | rs4072287 | A | C | 0.6059 | 0.095 | 0.017 | 2.29E-08 | 218735 | 0.004310073 | 946.8360596 |
|  | rs34783010 | T | G | 0.2566 | -0.1057 | 0.0191 | 3.13E-08 | 218735 | 0.004262449 | 936.329277 |
| Obesity due to excess calories | rs11030104 | G | A | 0.1665 | -0.1464 | 0.0266 | 3.72E-08 | 215767 | 0.005948836 | 1291.231911 |
|  | rs2270634 | C | T | 0.2341 | 0.1341 | 0.0232 | 7.46E-09 | 215767 | 0.006448535 | 1400.398587 |
|  | rs1121980 | A | G | 0.4284 | 0.2155 | 0.0199 | 2.50E-27 | 215767 | 0.022743968 | 5021.56241 |
|  | rs34783010 | T | G | 0.2567 | -0.1322 | 0.0227 | 5.75E-09 | 215767 | 0.006669341 | 1448.671996 |

**Supplementary Table 2. MR analyses of the causal effect of hypertension, diabetes, and obesity on tea intake**

| Trait |  | MR-Egger |  | WM |  | IVW |  | MR-PRESSO | Pleiotropy | Q | Heterogeneity |
| --- | --- | --- | --- | --- | --- | --- | --- | --- | --- | --- | --- |
|  |  | OR(95%CI) | *p* | OR(95%CI) | *p* | OR(95%CI) | *p* | Global Test *P* | *P* |  |  |
| Hypertension  Essential hypertension  Secondary hypertension  Diabetes  DM1  DM2  Obesity  Drug-induced obesity  Obesity due to excess calories | | 1.03(0.99-1.07) | 0.194 | 1.00(0.99-1.02) | 0.976 | 1.00(0.99-1.01) | 0.725 | 0.210 | 0.211 | 51.9 | 0.224 |
|  |  | 1.04(0.99-1.09) | 0.123 | 1.00(0.98-1.02) | 0.935 | 1.00(0.98-1.01) | 0.541 | 0.665 | 0.080 | 21.0 | 0.641 |
|  |  | NA | NA | NA | NA | NA | NA | NA | NA | NA | NA |
|  |  | 0.99(0.97-1.00) | 0.144 | 0.98(0.97-1.00) | 0.017 | 0.99(0.98-1.01) | 0.161 | 0.173 | 0.361 | 53.2 | 0.161 |
|  |  | 1.01(0.99-1.02) | 0.330 | 1.00(1.00-1.00) | 0.022 | 1.00(0.99-1.01) | 0.317 | 0.095 | 0.491 | 19.8 | 0.001 |
|  |  | 0.99(0.97-1.00) | 0.117 | 0.99(0.97-1.00) | 0.025 | 0.99(0.97-1.00) | 0.009 | 0.205 | 0.702 | 60.1 | 0.180 |
|  |  | 0.93(0.86-1.00) | 0.131 | 0.98(0.95-1.00) | 0.055 | 0.98(0.95-1.01) | 0.293 | 0.074 | 0.183 | 10.4 | 0.034 |
|  |  | NA | NA | NA | NA | NA | NA | NA | NA | NA | NA |
|  |  | 2.27(1.42-3.62) | 0.182 | 1.01(0.98-1.04) | 0.586 | 1.03(0.98-1.08) | 0.206 | NA | 0.188 | 10.8 | 0.005 |

**Supplementary Table 3. MR analyses of the causal effect of hypertension, diabetes, and obesity on green tea intake**

| Trait |  | MR-Egger |  | WM |  | IVW |  | MR-PRESSO | Pleiotropy | Q | Heterogeneity |
| --- | --- | --- | --- | --- | --- | --- | --- | --- | --- | --- | --- |
|  |  | OR(95%CI) | *p* | OR(95%CI) | *p* | OR(95%CI) | *p* | Global Test *P* | *P* |  |  |
| Hypertension  Essential hypertension  Secondary hypertension  Diabetes  DM1  DM2  Obesity  Drug-induced obesity  Obesity due to excess calories | | 0.72(0.12-4.33) | 0.720 | 0.71(0.34-1.50) | 0.367 | 0.93(0.56-1.56) | 0.796 | 0.645 | 0.766 | 50.07 | 0.627 |
|  |  | 1.01(0.10-10.43) | 0.992 | 0.68(0.28-1.66) | 0.395 | 0.95(0.51-1.77) | 0.872 | 0.466 | 0.956 | 32.53 | 0.490 |
|  |  | NA | NA | NA | NA | NA | NA | NA | NA |  | NA |
|  |  | 0.89(0.41-1.90) | 0.759 | 1.03(0.57-1.85) | 0.922 | 0.92(0.62-1.36) | 0.922 | 0.879 | 0.922 | 40.8 | 0.870 |
|  |  | 0.97(0.60-1.57) | 0.922 | 0.90(0.73-1.12) | 0.353 | 0.85(0.69-1.04) | 0.118 | 0.325 | 0.560 | 6.91 | 0.227 |
|  |  | 1.17(0.50-2.76) | 0.714 | 0.95(0.50-1.82) | 0.877 | 1.06(0.71-1.58) | 0.766 | 0.330 | 0.795 | 58.3 | 0.319 |
|  |  | 0.22(0.03-1.69) | 0.197 | 0.46(0.18-1.16) | 0.101 | 0.57(0.29-1.12) | 0.102 | 0.367 | 0.374 | 6.49 | 0.483 |
|  |  | NA | NA | NA | NA | NA | NA | NA | NA | NA | NA |
|  |  | 0.13(0.003-4.89) | 0.384 | 0.35(0.14-0.89) | 0.027 | 0.35(0.16-0.78) | 0.010 | 0.926 | 0.638 | 0.34 | 0.953 |

**Supplementary Table 4. MR analyses of the causal effect of hypertension, diabetes, and obesity on herbal tea intake**

| Trait |  | MR-Egger |  | WM |  | IVW |  | MR-PRESSO | Pleiotropy | Q | Heterogeneity |
| --- | --- | --- | --- | --- | --- | --- | --- | --- | --- | --- | --- |
|  |  | OR(95%CI) | *p* | OR(95%CI) | *p* | OR(95%CI) | *p* | Global Test *P* | *P* |  |  |
| Hypertension  Essential hypertension  Secondary hypertension  Diabetes  DM1  DM2  Obesity  Drug-induced obesity  Obesity due to excess calories | | 3.87(0.61-24.47) | 0.157 | 0.94(0.43-2.05) | 0.878 | 0.71(0.42-1.20) | 0.204 | 0.610 | 0.067 | 46.9 | 0.597 |
|  |  | 6.59(0.58-74.34) | 0.138 | 0.78(0.31-1.96) | 0.605 | 0.77(0.41-1.46) | 0.426 | 0.651 | 0.082 | 27.4 | 0.652 |
|  |  | NA | NA | NA | NA | NA | NA | NA | NA |  | NA |
|  |  | 0.67(0.32-1.44) | 0.310 | 0.90(0.50-1.62) | 0.721 | 0.77(0.52-1.14) | 0.198 | 0.501 | 0.678 | 52.1 | 0.472 |
|  |  | 1.28(0.88-1.86) | 0.271 | 0.91(0.74-1.12) | 0.369 | 0.90(0.73-1.10) | 0.301 | 0.375 | 0.106 | 7.5 | 0.188 |
|  |  | 0.76(0.34-1.70) | 0.504 | 0.58(0.31-1.07) | 0.080 | 0.83(0.57-1.21) | 0.323 | 0.559 | 0.814 | 51.8 | 0.559 |
|  |  | 0.65(0.09-4.75) | 0.683 | 1.01(0.44-2.31) | 0.978 | 1.08(0.56-2.11) | 0.816 | 0.873 | 0.610 | 3.0 | 0.882 |
|  |  | NA | NA | NA | NA | NA | NA | NA | NA | NA | NA |
|  |  | 1.51(0.04-55.15) | 0.844 | 0.98(0.40-2.43) | 0.966 | 0.86(0.39-1.91) | 0.716 | 0.862 | 0.785 | 0.8 | 0.834 |


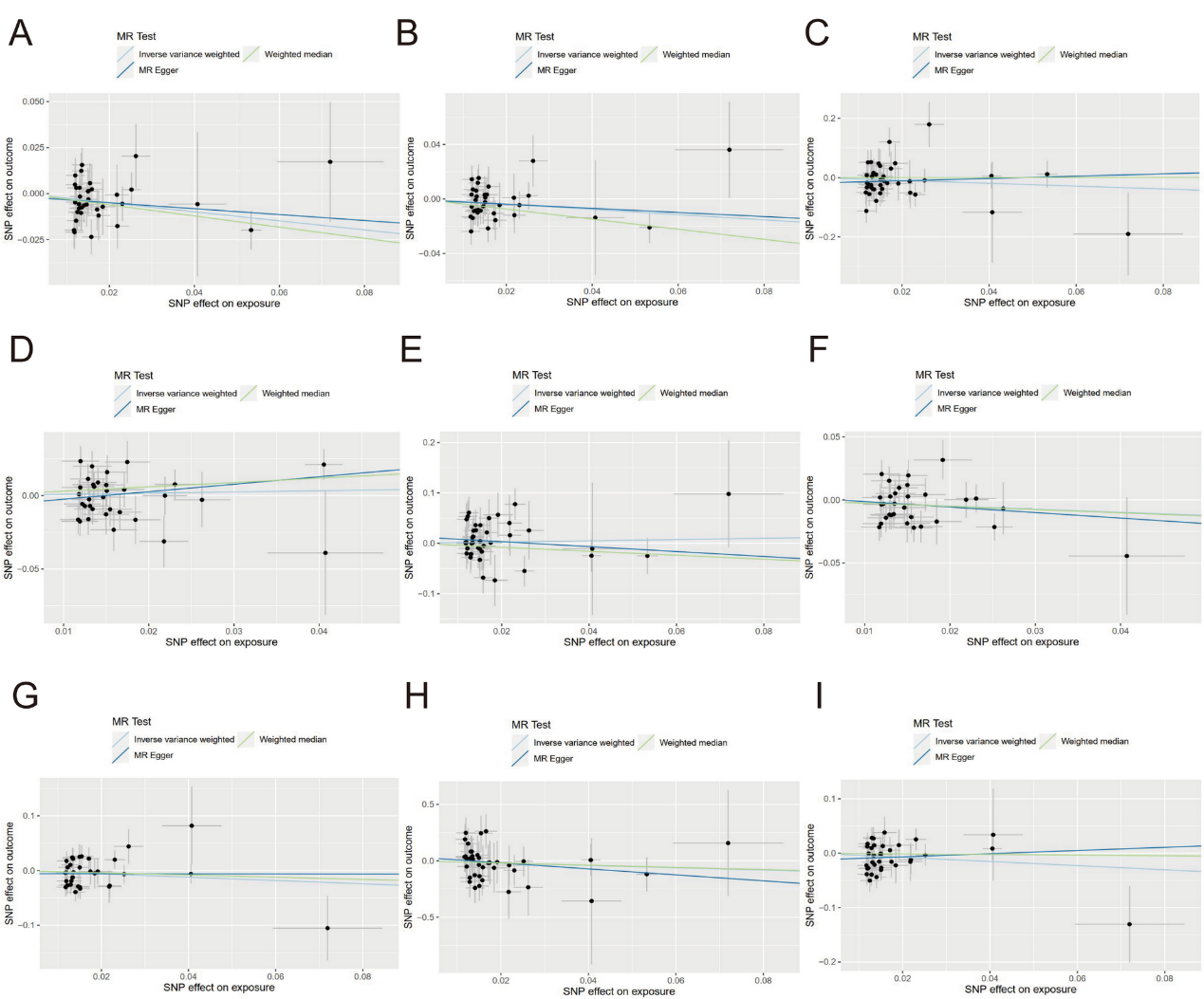


Supplementary Figure 1. Scatter plots for MR analyses of the causal effect of tea intake on hypertension, diabetes, and obesity. (A) hypertension; (B) essential hypertension; (C) secondary hypertension; (D) diabetes mellitus; (E) type 1 diabetes; (F) type 2 diabetes; (G) obesity; (H) obesity due to excess calories; (I) drug-induced obesity.


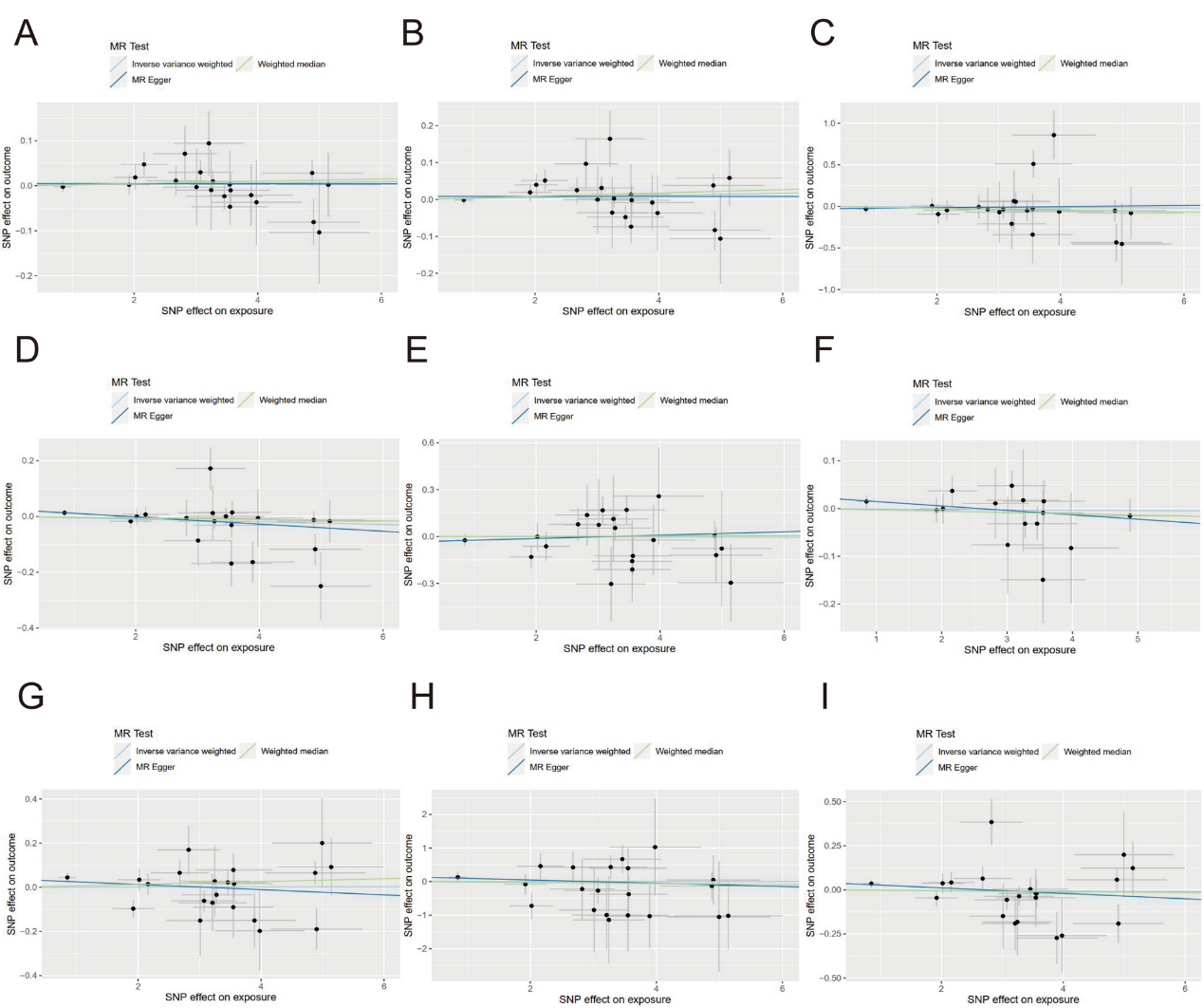


Supplementary Figure 2. Scatter plots for MR analyses of the causal effect of green tea intake on hypertension, diabetes, and obesity. (A) hypertension; (B) essential hypertension; (C) secondary hypertension; (D) diabetes mellitus; (E) type 1 diabetes; (F) type 2 diabetes; (G) obesity; (H) obesity due to excess calories; (I) drug-induced obesity.


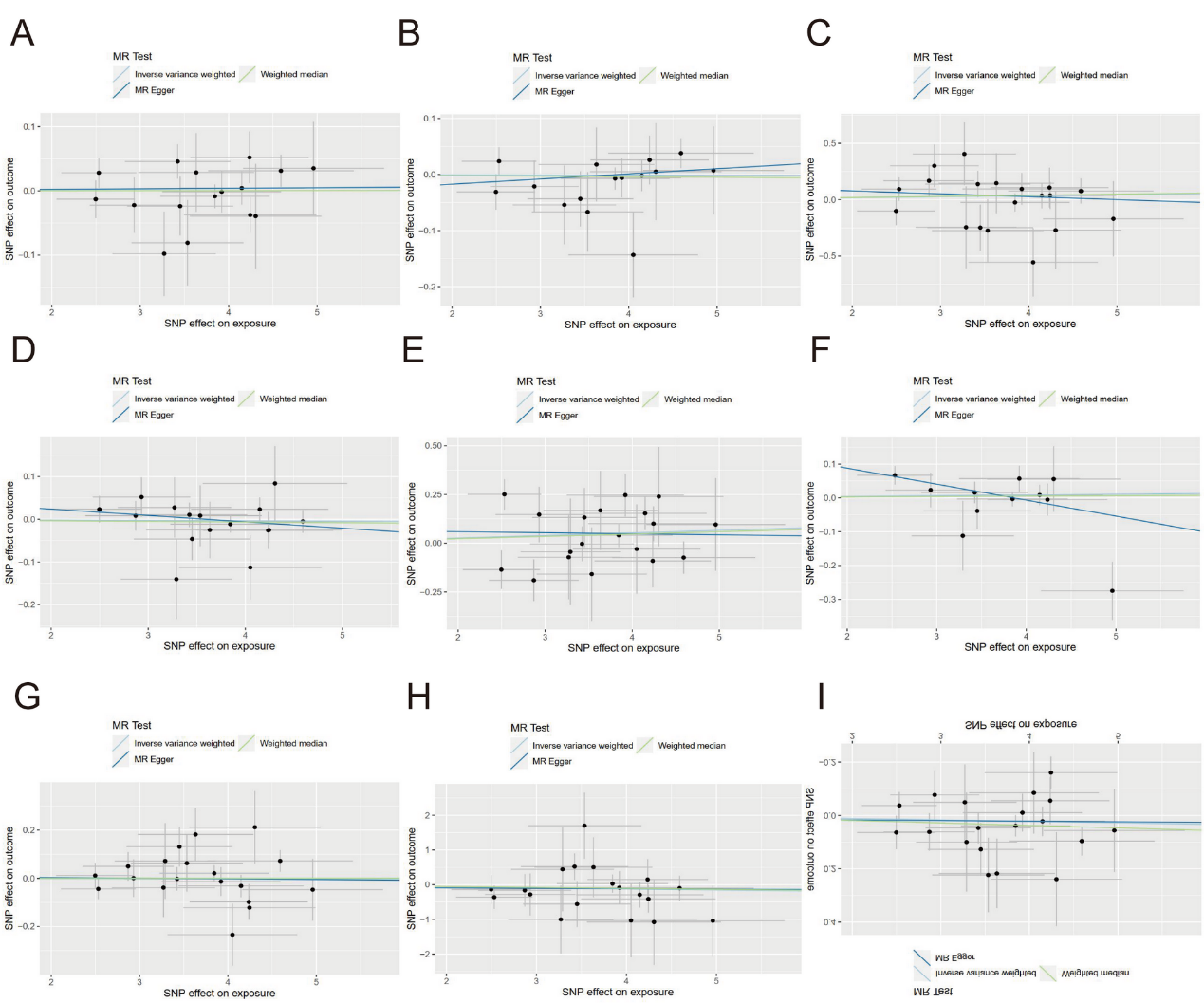


Supplementary Figure 3. Scatter plots for MR analyses of the causal effect of herbal tea intake on hypertension, diabetes, and obesity. (A) hypertension; (B) essential hypertension; (C) secondary hypertension; (D) diabetes mellitus; (E) type 1 diabetes; (F) type 2 diabetes; (G) obesity; (H) obesity due to excess calories; (I) drug-induced obesity.
